# Supplementary material for: Targeted Hybridization Capture of SARS-CoV-2 and Metagenomics Enables Genetic Variant Discovery and Nasal Microbiome Insights
Source: Microbiol Spectr. 2021 Sep 1;9(2):e00197-21. doi: 10.1128/Spectrum.00197-21 (PMC8557865; doi:10.1128/Spectrum.00197-21)
Supplement: SUPPLEMENTAL FILE 1 — Supplemental material. Download SPECTRUM00197-21_Supp_1_seq9.pdf, PDF file, 1.2 MB. [file spectrum00197-21_supp_1_seq9.pdf]

SUPPLEMENTARY MATERIALS

Targeted Hybridization Capture of SARS-CoV-2 and Metagenomics Enables Genetic Variant Discovery and Nasal Microbiome Insights

SARS-CoV-2 NGS Assay to Detect Genetic Variants

Dorottya Nagy-Szakal[a,b,†,#], Mara Couto-Rodriguez[a,†], Heather L. Wells[a], Joseph E. Barrows[a], Marilyne Debieu[a], Kristin Butcher[c], Siyuan Chen[c], Agnes Berki[d], Courteny Hager[a], Robert J. Boorstein[e], Mariah K. Taylor[f], Colleen B. Jonsson[f], Christopher E. Mason[a,g-j], Niamh B. O'Hara[a,b,k,#]

SUPPLEMENTARY DISCUSSION

Prospectives

The SARS-CoV-2 NGS Assay can be used as a method for viral detection by looking at data not only at an individual patient level, but on a population level as well. It also may be used to test the environment and determine how the SARS-CoV-2 virus is spreading in communities by sampling wastewater (1). This may be useful to track viral incidence and inform public health decisions on a community level. Due to shortages in COVID-19 diagnostic tests and testing sites, individuals are not always able to undergo regular testing, especially if they are not showing symptoms. Therefore, sampling wastewater can be used as a secondary option to determine how the virus fluctuates in the community and directs public health measurements to limit the spread of the virus.

Hybrid capture NGS-based methods are also a powerful approach to anticipate and characterize emerging human viruses transmitted from wildlife. NGS enables characterization of genes and pathogenicity important in transmission from animals to humans. Importantly, genetic similarity to known pathogens in PCR-targeted regions of the genome is not an accurate predictor of human pathogenicity (2–4). Coronaviruses, in particular, cannot be accurately confirmed or dismissed as human pathogens based on these PCR fragments because of the propensity for coronavirus genomes to recombine, particularly in the spike gene (5). NGS provides a valuable tool for the detection of emerging viruses in domestic animals and wildlife, and generates critical data that is needed to characterize the potential for a virus to be pathogenic in humans. Several studies have demonstrated the potential of animal reservoirs of SARS-CoV-2 such as cats, dogs, bats, minks among others (6). Surveillance of animal reservoirs using the presented assay will not only add to our understanding of SARS-CoV-2 adaptation during

infection of animal hosts but could potentially identify viruses similar to SARS-CoV-2 with pandemic potential.

Limitations and future work

Our study findings were limited by the cross-sectional nature of testing. While the current work highlights the importance of the characterization of the SARS-CoV-2 viral genome and provides valuable insights into the genetic variants and geographic origin of variants, extension of our work is needed by processing additional samples collected from additional geographic regions and with additional clinical metadata that could enable future risk stratification linked to novel variants. Additional longitudinal testing of SARS-CoV-2-positive samples using our technology could enable further screening of variants in the same individual over time to monitor intra-host variation and evolution, as well as within populations in the same geographic location. Further optimization is needed to accommodate different sample types (saliva and other respiratory specimens) and to extend our variant curation to support the continuously growing scientific knowledge around genetic variants and their role in viral fitness. While we observed intra-host diversity through visualizing sequence alignments, our variant caller only reports the most frequent nucleotide or variant at each genomic site. Incorporating calling of multiple variants when present at each genome site to provide intra-host diversity could prove useful.

Although our work contributes to the ongoing elucidation of the role of the microbiome and secondary infections commonly associated with COVID-19, further longitudinal studies linking disease outcome to microbial load and dynamics are required for better understanding the virus, as well as microbe and host interactions during the course of the disease. SARS-CoV-2 negative samples should not be considered healthy since clinical metadata related to any

respiratory symptoms have not been reported at the time of collection. Expanding the cohort and collecting clinical metadata will be a key next step since it creates an unprecedented opportunity to map the genetic characterization of the virus and patient history (reason of sampling, travel history, co-morbidities, prior medication) with disease outcome, disease progression, and hospitalization risk.

SUPPLEMENTARY METHODS

Orthogonal RT-qPCR technology

RT-qPCR technology (Panther Fusion SARS-CoV-2 Assay [Hologic, Marlborough, MA]; and cobas ® SARS-CoV-2 test with the Cobas 6800/8800 System [Roche, Basel, Switzerland]) was used to define the presence of SARS-CoV-2 viral RNA (SARS-CoV-2-positive: CVP and SARS-CoV-2-negative: CVN; **Table S4**). These technologies have been issued EUA approval by the FDA for the clinical diagnostics of COVID-19. The CT value was defined using GenArraytion COVID-19 duplex RT-qPCR (Rockville, MD) and cobas ® SARS-CoV-2 test with the Cobas 6800/8800 System (Roche, Basel, Switzerland) (**Table S4**).

Quality Control

Quality control steps were performed after nucleic acid extraction, cDNA synthesis, cDNA library generation, target enrichment, metagenomic library, and final sequencing pools. These steps included analyses to determine nucleic acid concentration and fragment size using Qubit RNA High Sensitivity/ dsDNA High Sensitivity/ dsDNA Broad Range Quantitation Assay (Thermo Fisher Scientific, Waltham, MA) and TapeStation D5000/ D1000/ D1000 High Sensitivity platform (Agilent, Santa Clara, CA).

Inclusivity and Exclusivity Study Design

To evaluate the inclusivity of the assay to capture different SARS-CoV-2 lineages, we processed six synthetic control samples created by Twist Bioscience with known genetic sequences (Twist Bioscience control 1-6, MT007544.1, MN908947.3, LC528232.1, MT106054.1, MT188340, MT118835), including the original Wuhan coronavirus strain, through laboratory processing and the bioinformatics pipeline.

The synthetic controls contain the following variants: control 1: T19065C, T22303G, G26144T, ACGATCGAGTG29749A; control 3: TG11082T; control 4: C9924T; control 5: T514C, C17410T; control 6: C8782T, T18603C, T18975A, A19175C, C27925T, T28144C, C29095T. We extended our validation for the novel UK B.1.1.7_710528 (with mutations including C240T, C912T, C3036T, C3266T, C5387A, C5985T, T6953C, 11287delTCTGGTTTT, C14407T, C14675T, C15278T, C15856T, T16175C, A17614G, 21764delTACATG, 21990delTTA, A23062T, C23270A, A23402G, C23603A, C23708T, T24505G, G24913C, T27884C, C27971T, G28047T, A28110G, 28270delA, G28279C, A28280T, T28281A, G28880A, G28881A, G28882C, and C28976T), the UK B.1.1.7_601443 (with mutations including C240T, C912T, C3036T, C3266T, C5387A, C5985T, T6953C, 11287delTCTGGTTTT, C14407T, C14675T, C15278T, T16175C, 21764delTACATG, 21990delTTA, A23062T, C23270A, A23402G, C23603A, C23708T, T24505G, G24913C, C27971T, G28047T, A28110G, G28279C, A28280T, T28281A, G28880A, G28881A, G28882C and C28976T) and South African EPI_ISL_678597 strain (with mutations including G173T, C240T, C1058T, A2691T, C3036T, G5229T, A10322G, 11287delTCTGGTTTT, C14407T, A21800C, A22205G, 22280delCTTTACTTG, G22812T, G23011A, A23062T, A23402G, C23663T, G25562T, C25903T, C26455T, C28252T, A28253C, C28886T, G29556T).

We also performed the following in-silico inclusivity analysis. First, we downloaded 151,323 high-quality SARS-CoV-2 viral genome nucleotide sequences from GISAID (3/29/2021) in FASTA format. To enable efficient computational evaluation of these data, we indexed the combined FASTAs and split them into subsets for parallelization. We performed BLASTn alignments using all 994 probes found in the SARS-CoV-2 Research Panel, with the PolyA tail removed, against the parallelized database. We similarly eliminated the PolyA tail from alignment in our pipeline. We chose to present descriptive statistics of the data due to the breadth of high identity matches to the SARS-CoV-2 viral genome.

To account for potential cross-reactivity of probe sequences found in the SARS-CoV-2 Research Panel, we aligned reads to 30 microbial genomes and the human genome along with the SARS-CoV-2 viral genome downloaded from NCBI (**Table S5**). A BLASTn (NCBI) analysis was then performed to quantify the number of primer pairs that had more than 80% homology with each of the genomes in the cohort. Then, we extended our in-silico exclusivity analysis using approximately 3.6 million viral nucleotide sequences from NCBI Virus (2/16/2021; FASTA format), representing the entire available database of viral nucleotide sequences.

Analytical Sensitivity of the SARS-CoV-2 NGS Assay

The analytical sensitivity (Limit of Detection [LoD]) study established the lowest concentration of SARS-CoV-2 viral genome (copies/ml) that can be detected by the SARS-CoV-2 NGS Assay at least 95% of the time. The preliminary LoD was established by testing 10-fold dilutions of SARS-CoV-2 synthetic RNA (MN908947.3, Twist Bioscience, #102024). The preliminary LoD was confirmed by testing triplicates of 2-fold dilutions (2560 copies/ml, 1280

copies/ml, 640 copies/ml, 320 copies/ml, 160 copies/ml, 80 copies/ml, and 40 copies/ml) by spiking the quantified heat-inactivated SARS-CoV-2 (ATCC, VR-1986HK) into negative respiratory clinical matrices (NP swabs -clinical samples previously tested negative for SARS-CoV-2 RNA). Then, the LoD was replicated 30 times.

Bioinformatic Pipeline of the SARS-CoV-2 NGS Assay

Preprocessing of Sequencing Data

The program cutadapt trimmed adapter sequences using the standard Illumina adapter sequence "AGATCGGAAGAGC" and a minimum read length of 20bp. Samples with higher than 50,000,000 reads were subsampled to 50,000,000 reads using seqtk before further analysis. FASTQs were split for parallelization and aligned against the genomes of SARS-CoV-2 virus (NC_045512.2), 26 other viral and bacterial respiratory pathogens, and the human genome using Bowtie2 run in local mode. The algorithm to detect SARS-CoV-2 used only reads that map unambiguously to the SARS-CoV-2 reference (NC_045512.2) in order to minimize false-positives due to cross-hybridization of the probes or cross-contamination during the Twist NGS hybrid capture. Unambiguously mapped reads were extracted using samtools-view. Duplicates and read statistics were calculated with Picard (2.23.0) (7) and the CollectHSMetrics function. The depth of reads at each site across the SARS-CoV-2 genome is calculated using samtools-depth. Test samples and/or positive controls with fewer than 10,000 total reads are assigned as invalid.

Presence/absence calculation and coverage

Detection of the SARS-CoV-2 virus is based on the degree to which the genome was recovered in sequencing. The more genome that is recovered, the more likely it is that the virus

is present. A sample-specific integral is computed by calculating the coverage at 1X depth using a sliding window scheme (with a window size of 1000 and step size of 100). This metric is then log transformed. A threshold of 8.6 for samples with less than or equal to 10,000 bases on-target or 9.6 for samples with more than 10,000 bases on-target determines if SARS-CoV-2 is present or absent. The integral is calculated using the R statistical software package (v4.0.1).

Genetic Variants Detection and Phylogenetic Analysis

The BAM file was subsampled to a maximum of 500X coverage at a given site within the SARS-CoV-2 viral genome because coverage can exceed 8,000X, which is computationally prohibitive for variant calling tools. Picard MarkDuplicates was used to mark and remove PCR duplicates. Variants were called in the subsampled BAM file using GATK GenotypeVCF and HaplotypeCaller. Variants were filtered by minimum depth (10 reads) using annotate-vcf. A consensus genome from the filtered VCF and NC_045512.2 was created using GATK FastaAlternateReferenceMaker. Command-line Nextclade (04/22/2021) was used to identify the clade and generate a phylogenetic tree. The tree was displayed using a modified version of BioPhylo.

The genetic variant annotation provides information on the mutation name, gene location, protein name, amino acid change (NCBI), and synonymy. Additional information included the frequency of genetic variants in our sample cohort. The Grantham scoring system was used to designate conservative and radical mutations. In this system, the score of 100 and above calls mutations radical.

To validate the variant calling of our assay and software, we selected 3 genetic regions containing mutations identified by our protocol (**Table S7A**). The genetic regions were PCR amplified using specifically designed primers followed by Sanger sequencing. The list of

validated genetic variants and the primer sets are described in the supplemental table (**Table S7A**). The primer design considered nearby mutations to the selected variants in order to maximize the validation efficiency of the sequencing reaction.

Internal Controls

Viral transport media (VTM) was used as a negative/no template control (NTC) to eliminate the possibility of sample contamination on the assay run and was used with each extraction batch through sequencing. VERO E6 cells [ATCC CRL-1586] spiked into VTM were used as a negative extraction control (NEC) to monitor for any cross-contamination that occurs during the extraction process, as well as an extraction control to validate extraction reagents and successful RNA extraction. Positive Twist RNA template control - synthetic SARS-CoV-2 RNA Control 2 (MN908947.3) in Gene Expression Universal Reference RNA (Human, Agilent) was used as a positive control (PC) to verify that the assay run was performing as intended. The PC is made of six RNA fragments that are 5000bp in length spanning the entire SARS-CoV-2 viral genome (MN908947.3). All coding and non-coding regions of the viral genome are included in the PC, except for the polyadenylated region. Each RNA fragment is made by *in vitro* transcription using the DNA template as reference. Gene Expression Universal Reference RNA (Human, Agilent) was used as an internal control (IC) to validate the SARS-CoV-2 NGS Assay reagents and successful library generation. Additionally, the number of reads mapped to the human genome was determined and used as internal control in each specimen.

Metagenomic Validation

Since *Prevotella spp*. were found to be a biomarker for CVP status based on the data obtained by two independent bioinformatic pipelines but were found to conflict at the species-level classification, we used 16s rDNA sequencing to aid in confirming the phylogeny of the correct *Prevotella* species. MetaPhlAn2 classified the biomarker as *Prevotella salivae* (LDA score: 3.77) while BIOTIA-DX called it as *Prevotella oral taxon 299* (LDA score: 3.81). For primer design, *Prevotella spp*. type strain 16s rDNA sequences from the ribosomal database project (RDP) (8), including *Prevotella sp. oral taxon 299*, were aligned. We modified Zozaya-Hinchliffe et al. (9) forward primer to add degeneracy and designed a reverse primer that would yield ~1200bp of the 16s rDNA (**Table S8B**). The primer set was then tested for *in-silico* specificity against the curated 16s rDNA dataset from RDP and was found to be specific to 29/44 *Prevotella spp.,* out of the 13,324 16S rDNA type strain sequences present in the database, including those of interest to this study. The PCR amplification condition and primer set are described in **Table S8B**. PCR amplicons were excised from a modified-TAE agarose gel and purified with Millipore Ultrafree™-DA Centrifugal Filter Device, cloned, and sent for Sanger sequencing with T7/SP6 primers in a reference laboratory. Vector sequences were trimmed from 16s rDNA sequences, then sequences were aligned with all *Prevotella spp*. type strains. Kimura-2-parameter neighbor-joining phylogenetic tree with bootstrap of 500 was constructed with MegaX.

REFERENCES

1. Hart OE, Halden RU. 2020. Computational analysis of SARS-CoV-2/COVID-19 surveillance by wastewater-based epidemiology locally and globally: Feasibility, economy, opportunities and challenges. Sci Total Environ 730.
2. Goldstein T, Anthony SJ, Gbakima A, Bird BH, Bangura J, Tremeau-Bravard A, Belaganahalli MN, Wells HL, Dhanota JK, Liang E, Grodus M, Jangra RK, DeJesus VA, Lasso G, Smith BR, Jambai A, Kamara BO, Kamara S, Bangura W, Monagin C, Shapira S, Johnson CK, Saylors K, Rubin EM, Chandran K, Lipkin WI, Mazet JAK. 2018. The discovery of Bombali virus adds further support for bats as hosts of ebolaviruses. Nat Microbiol 3:1084–1089.
3. Marsh GA, de Jong C, Barr JA, Tachedjian M, Smith C, Middleton D, Yu M, Todd S, Foord AJ, Haring V, Payne J, Robinson R, Broz I, Crameri G, Field HE, Wang LF. 2012. Cedar Virus: A Novel Henipavirus Isolated from Australian Bats. PLoS Pathog 8.
4. Ren W, Qu X, Li W, Han Z, Yu M, Zhou P, Zhang S-Y, Wang L-F, Deng H, Shi Z. 2008. Difference in Receptor Usage between Severe Acute Respiratory Syndrome (SARS) Coronavirus and SARS-Like Coronavirus of Bat Origin. J Virol 82:1899–1907.
5. Wells HL, Letko M, Lasso G, Ssebide B, Nziza J, Byarugaba DK, Macias NI, Liang E, Cranfield M, Han BA, Tingley MW, Diuk-Wasser M, Goldstein T, Johnson CK, Mazet J, Chandran K, Munster VJ, Gilardi K, Anthony SJ. 2020. The evolutionary history of ACE2 usage within the coronavirus subgenus Sarbecovirus. bioRxiv.
6. Delahay RJ, de la Fuente J, Smith GC, Sharun K, Snary EL, Flores Girón L, Nziza J, Fooks AR, Brookes SM, Lean FZX, Breed AC, Gortazar C. Assessing the risks of SARS-CoV-2 in wildlife. One Health Outlook. 2021;3:7. eCollection 2021.

7. Broad Institute. 2009. Picard Tools - By Broad Institute. Github.

8. McArthur AG, Waglechner N, Nizam F, Yan A, Azad MA, Baylay AJ, Bhullar K, Canova MJ, De Pascale G, Ejim L, Kalan L, King AM, Koteva K, Morar M, Mulvey MR, O'Brien JS, Pawlowski AC, Piddock LJV, Spanogiannopoulos P, Sutherland AD, Tang I, Taylor PL, Thaker M, Wang W, Yan M, Yu T, Wright GD. 2013. The comprehensive antibiotic resistance database. Antimicrob Agents Chemother 57:3348–3357.

9. Zozaya-Hinchliffe M, Lillis R, Martin DH, Ferris MJ. 2010. Quantitative PCR assessments of bacterial species in women with and without bacterial vaginosis. J Clin Microbiol 48:1812–1819.

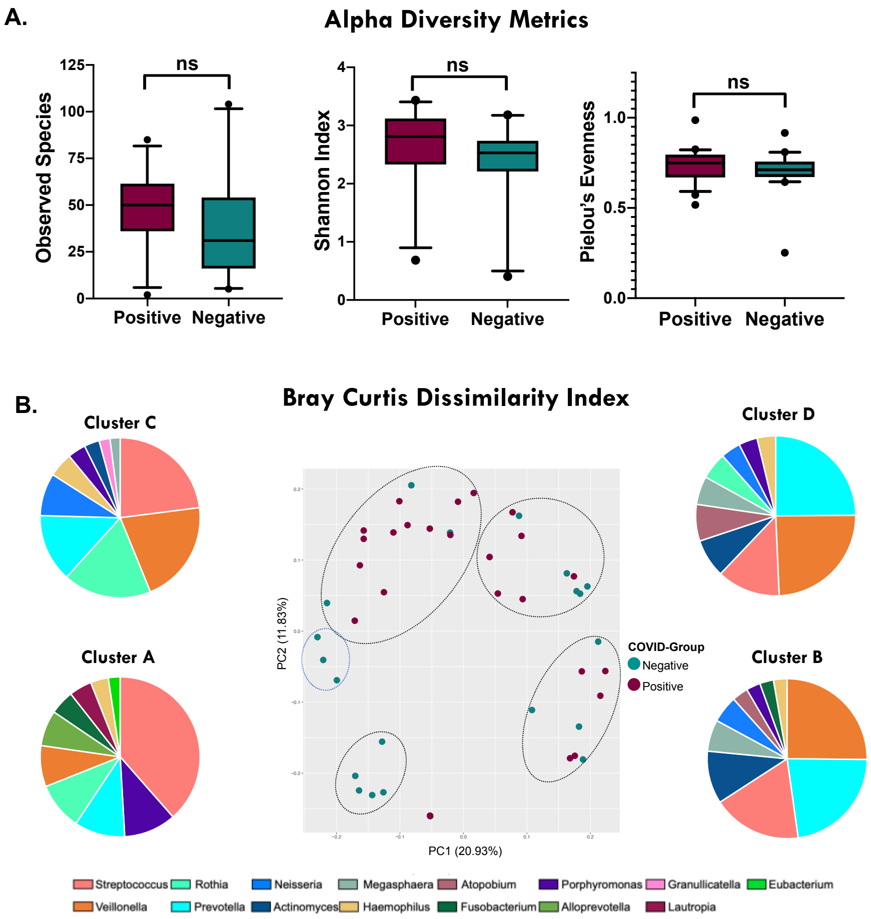

Fig. S1. Nasopharyngeal microbiome profiles are not driven by COVID-status. (A) Alpha diversity metrics show no difference in microbiome richness or evenness based on COVID status. Observed Species, Shannon Index and Pielou's Evenness were not significant based on a Welch's t-test comparing CVP (n= 26) and CVN (n = 22). (B) Bray Curtis Dissimilarity Index exhibited four clusters (A, B, C, and D) with most of the separation driven by *Rothia mucilaginosa* along PC1 (20.9%) and clustering along. PC2 (11.83%) is explained by the presence/absence of *Streptococcus parasanguinis* and unclassified *Neisseria*. Pie charts represent the top 10 most abundant bacterial genera among samples within each cluster.

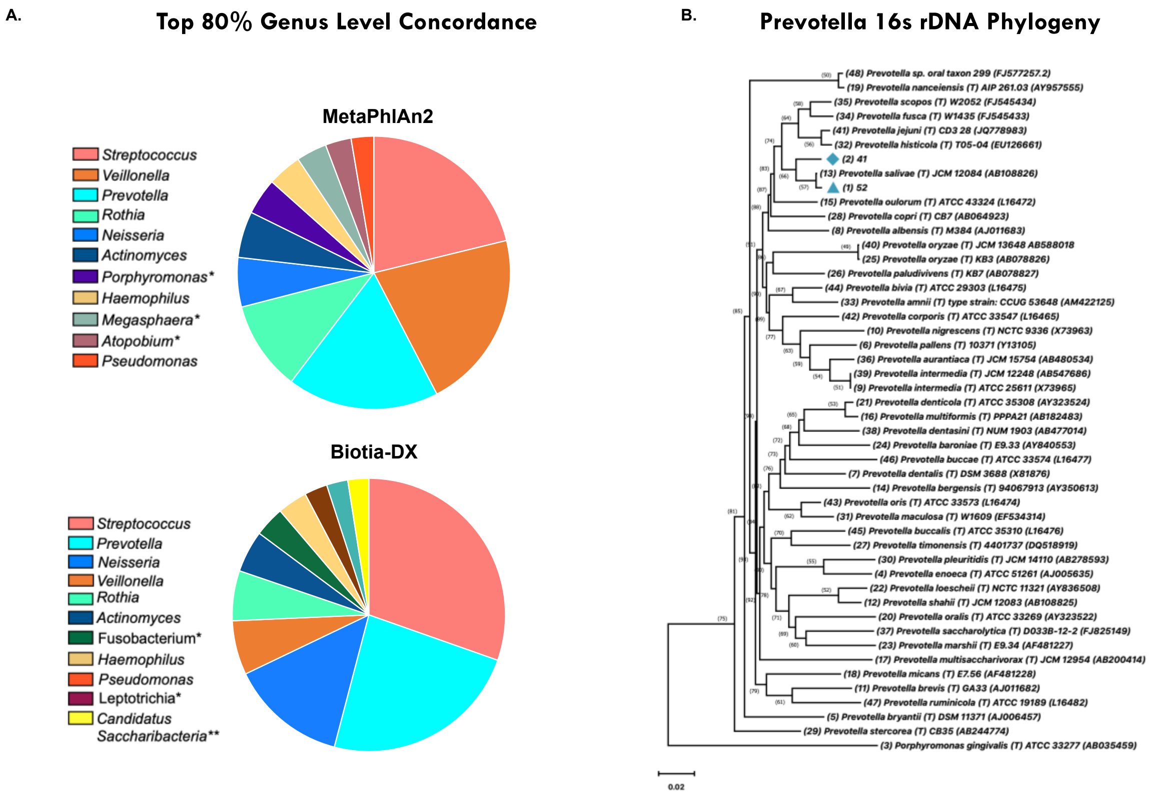

Fig. S2. Comparison bioinformatic pipelines exhibit high concordance in the classification of the nasopharyngeal microbiome. (A) Pie charts depicting the top 80% relative abundance of bacteria at the genus level for MetaPhlAn2 and BIOTIA-DX classification. (B) Neighbor joining phylogenetic tree of partial Prevotella spp.16s rDNA confirms the Prevotella species increased in CVP samples is closely related to Prevotella salivae. The optimal tree with the sum of branch length (=1.65) is shown. The percentage of replicate trees in which the associated taxa clustered together in the bootstrap test (500 replicates) is shown next to the branches. The tree is drawn to scale, with branch lengths in the same units as those of the evolutionary distances used to infer the phylogenetic tree. The evolutionary distances were computed using the Kimura-2-parameter method and are in the units of the number of base substitutions per site. This analysis involved 48 nucleotide sequences. All ambiguous positions were removed for each sequence pair (pairwise deletion option). There were a total of 997 positions in the final dataset. Evolutionary analyses were conducted in MEGA X.

Supplemental Table 1. Specimen information of 140 samples used in the validation study, independent validation and geographic study with specification on the collection device, viral transport media, the comparator RT-PCR methodology, results and
VTM: viral transport media; NA: we were not able to identify the collection device and matrix due to the label damage during de-identification of samples.

Specimen ID	Collection Device	Matrix (VTM)	Study	Collection Date	Location (State)	Comparator RT-PCR Results	Comparator RT-PCR Methodology	Mean_Ct_value using GenArraytion	Mean_Ct_value using Roche Cobas (ORF1 gene)	Mean_Ct_value using Roche Cobas (E gene)	Biotia ID
CVN-1	BD	UTM	Validation Study	April	New York	NEGATIVE	Panther Fusion	Undetermined	NA	NA	COVID-NEG-002
CVN-2	PURITAN	UTM	Validation Study	April	New York	NEGATIVE	Panther Fusion	Undetermined	NA	NA	COVID-NEG-003
CVN-3	STARPLEX	MULTITRANS	Validation Study	April	New York	NEGATIVE	Panther Fusion	Not enough sample	NA	NA	COVID-NEG-004
CVN-4	PURITAN	UTM	Validation Study	April	New York	NEGATIVE	Panther Fusion	Not enough sample	NA	NA	COVID-NEG-005
CVN-5	PURITAN	UTM	Validation Study	April	New York	NEGATIVE	Panther Fusion	Undetermined	NA	NA	COVID-NEG-006
CVN-6	COPAN	UTM	Validation Study	April	New York	NEGATIVE	Panther Fusion	Undetermined	NA	NA	COVID-NEG-007
CVN-7	COPAN	UTM	Validation Study	April	New York	NEGATIVE	Panther Fusion	Not enough sample	NA	NA	COVID-NEG-008
CVN-8	STARPLEX	MULTITRANS	Validation Study	April	New York	NEGATIVE	Panther Fusion	Not enough sample	NA	NA	COVID-NEG-009
CVN-9	PURITAN	UTM	Validation Study	April	New York	NEGATIVE	Panther Fusion	Undetermined	NA	NA	COVID-NEG-010
CVN-10	BD	UTM	Validation Study	April	New York	NEGATIVE	Panther Fusion	Undetermined	NA	NA	COVID-NEG-011
CVN-11	PURITAN	UTM	Validation Study	April	New York	NEGATIVE	Panther Fusion	Undetermined	NA	NA	COVID-NEG-012
CVN-12	BD	UTM	Validation Study	April	New York	NEGATIVE	Panther Fusion	Undetermined	NA	NA	COVID-NEG-013
CVN-13	NA	NA	Validation Study	April	New York	NEGATIVE	Panther Fusion	Undetermined	NA	NA	COVID-NEG-014
CVN-14	STARPLEX	MULTITRANS	Validation Study	April	New York	NEGATIVE	Panther Fusion	Undetermined	NA	NA	COVID-NEG-015
CVN-15	COPAN	UTM	Validation Study	April	New York	NEGATIVE	Panther Fusion	Undetermined	NA	NA	COVID-NEG-016
CVN-16	COPAN	UTM	Validation Study	April	New York	NEGATIVE	Panther Fusion	Undetermined	NA	NA	COVID-NEG-017
CVN-17	BD	UTM	Validation Study	April	New York	NEGATIVE	Panther Fusion	Undetermined	NA	NA	COVID-NEG-018
CVN-18	BD	UTM	Validation Study	April	New York	NEGATIVE	Panther Fusion	Undetermined	NA	NA	COVID-NEG-019
CVN-19	BD	UTM	Validation Study	April	New York	NEGATIVE	Panther Fusion	Undetermined	NA	NA	COVID-NEG-020
CVN-20	PURITAN	UTM	Validation Study	April	New York	NEGATIVE	Panther Fusion	Undetermined	NA	NA	COVID-NEG-021
CVN-21	BD	UTM	Validation Study	April	New York	NEGATIVE	Panther Fusion	Undetermined	NA	NA	COVID-NEG-022
CVN-22	BD	UTM	Validation Study	April	New York	NEGATIVE	Panther Fusion	Undetermined	NA	NA	COVID-NEG-023
CVN-23	STARPLEX	MULTITRANS	Validation Study	April	New York	NEGATIVE	Panther Fusion	Undetermined	NA	NA	COVID-NEG-024
CVN-24	BD	UTM	Validation Study	April	New York	NEGATIVE	Panther Fusion	Undetermined	NA	NA	COVID-NEG-025
CVN-25	BD	UTM	Validation Study	April	New York	NEGATIVE	Panther Fusion	Undetermined	NA	NA	COVID-NEG-026
CVN-26	BD	UTM	Validation Study	April	New York	NEGATIVE	Panther Fusion	Undetermined	NA	NA	COVID-NEG-027
CVN-27	BD	UTM	Validation Study	April	New York	NEGATIVE	Panther Fusion	Undetermined	NA	NA	COVID-NEG-028
CVN-28	BD	UTM	Validation Study	April	New York	NEGATIVE	Panther Fusion	Undetermined	NA	NA	COVID-NEG-029
CVN-29	BD	UTM	Validation Study	April	New York	NEGATIVE	Panther Fusion	Undetermined	NA	NA	COVID-NEG-030
CVN-30	BD	UTM	Validation Study	April	New York	NEGATIVE	Panther Fusion	Undetermined	NA	NA	COVID-NEG-031
CVP-1	REMEL M6	M6	Validation Study	April	New York	POSITIVE	Panther Fusion	27.230	NA	NA	COVID-POS-006
CVP-2	COPAN	UTM	Validation Study	April	New York	POSITIVE	Panther Fusion	24.713	NA	NA	COVID-POS-007
CVP-3	REMEL M6	M6	Validation Study	April	New York	POSITIVE	Panther Fusion	26.727	NA	NA	COVID-POS-008
CVP-4	REMEL M6	M6	Validation Study	April	New York	POSITIVE	Panther Fusion	31.122	NA	NA	COVID-POS-009
CVP-5	BD	UTM	Validation Study	April	New York	POSITIVE	Panther Fusion	29.452	NA	NA	COVID-POS-010
CVP-6	STARPLEX	MULTITRANS	Validation Study	April	New York	POSITIVE	Panther Fusion	15.660	NA	NA	COVID-POS-011
CVP-7	BD	UTM	Validation Study	April	New York	POSITIVE	Panther Fusion	Undetermined	NA	NA	COVID-POS-012
CVP-8	BD	UTM	Validation Study	April	New York	POSITIVE	Panther Fusion	Undetermined	NA	NA	COVID-POS-013
CVP-9	BD	UTM	Validation Study	April	New York	POSITIVE	Panther Fusion	24.863	NA	NA	COVID-POS-014
CVP-10	COPAN	UTM	Validation Study	April	New York	POSITIVE	Panther Fusion	33.331	NA	NA	COVID-POS-015
CVP-11	COPAN	UTM	Validation Study	April	New York	POSITIVE	Panther Fusion	Undetermined	NA	NA	COVID-POS-016
CVP-12	REMEL M6	M6	Validation Study	April	New York	POSITIVE	Panther Fusion	19.900	NA	NA	COVID-POS-017
CVP-13	COPAN	UTM	Validation Study	April	New York	POSITIVE	Panther Fusion	Undetermined	NA	NA	COVID-POS-018
CVP-14	BD	UTM	Validation Study	April	New York	POSITIVE	Panther Fusion	Undetermined	NA	NA	COVID-POS-019
CVP-15	REMEL M6	M6	Validation Study	April	New York	POSITIVE	Panther Fusion	21.450	NA	NA	COVID-POS-020
CVP-16	BD	UTM	Validation Study	April	New York	POSITIVE	Panther Fusion	Undetermined	NA	NA	COVID-POS-021
CVP-17	BD	UTM	Validation Study	April	New York	POSITIVE	Panther Fusion	30.931	NA	NA	COVID-POS-022
CVP-18	BD	UTM	Validation Study	April	New York	POSITIVE	Panther Fusion	32.486	NA	NA	COVID-POS-023
CVP-19	BD	UTM	Validation Study	April	New York	POSITIVE	Panther Fusion	24.077	NA	NA	COVID-POS-024
CVP-20	BD	UTM	Validation Study	April	New York	POSITIVE	Panther Fusion	32.812	NA	NA	COVID-POS-025
CVP-21	BD	UTM	Validation Study	April	New York	POSITIVE	Panther Fusion	29.486	NA	NA	COVID-POS-026
CVP-22	BD	UTM	Validation Study	April	New York	POSITIVE	Panther Fusion	20.357	NA	NA	COVID-POS-027
CVP-23	NA	NA	Validation Study	April	New York	POSITIVE	Panther Fusion	22.050	NA	NA	COVID-POS-028
CVP-24	COPAN	UTM	Validation Study	April	New York	POSITIVE	Panther Fusion	24.973	NA	NA	COVID-POS-029
CVP-25	COPAN	UTM	Validation Study	April	New York	POSITIVE	Panther Fusion	22.371	NA	NA	COVID-POS-030
CVP-26	BD	UTM	Validation Study	April	New York	POSITIVE	Panther Fusion	28.781	NA	NA	COVID-POS-031
CVP-27	BD	UTM	Validation Study	April	New York	POSITIVE	Panther Fusion	33.635	NA	NA	COVID-POS-032
CVP-28	COPAN	UTM	Validation Study	April	New York	POSITIVE	Panther Fusion	17.872	NA	NA	COVID-POS-033
CVP-29	BD	UTM	Validation Study	April	New York	POSITIVE	Panther Fusion	33.757	NA	NA	COVID-POS-034
CVP-30	COPAN	UTM	Validation Study	April	New York	POSITIVE	Panther Fusion	31.576	NA	NA	COVID-POS-035
CVN-31	Aptima MultiTest	STM	Independent Vali	July	New York	NEGATIVE	Panther Fusion	Undetermined	NA	NA	COVID-NEG-032
CVN-32	Aptima MultiTest	STM	Independent Vali	July	New York	NEGATIVE	Panther Fusion	Undetermined	NA	NA	COVID-NEG-033
CVN-33	Aptima MultiTest	STM	Independent Vali	July	New York	NEGATIVE	Panther Fusion	Undetermined	NA	NA	COVID-NEG-034
CVN-34	Aptima MultiTest	STM	Independent Vali	July	New York	NEGATIVE	Panther Fusion	Undetermined	NA	NA	COVID-NEG-035
CVN-35	Aptima MultiTest	STM	Independent Vali	July	New York	NEGATIVE	Panther Fusion	Undetermined	NA	NA	COVID-NEG-036
CVN-36	Aptima MultiTest	STM	Independent Vali	July	New York	NEGATIVE	Panther Fusion	Undetermined	NA	NA	COVID-NEG-037
CVN-37	Aptima MultiTest	STM	Independent Vali	July	New York	NEGATIVE	Panther Fusion	Undetermined	NA	NA	COVID-NEG-038
CVN-38	Aptima MultiTest	STM	Independent Vali	July	New York	NEGATIVE	Panther Fusion	Undetermined	NA	NA	COVID-NEG-039
CVN-39	Aptima MultiTest	STM	Independent Vali	July	New York	NEGATIVE	Panther Fusion	Undetermined	NA	NA	COVID-NEG-040
CVN-40	Aptima MultiTest	STM	Independent Vali	July	New York	NEGATIVE	Panther Fusion	Undetermined	NA	NA	COVID-NEG-041
CVN-41	Aptima MultiTest	STM	Independent Vali	July	New York	NEGATIVE	Panther Fusion	Undetermined	NA	NA	COVID-NEG-042
CVN-42	Aptima MultiTest	STM	Independent Vali	July	New York	NEGATIVE	Panther Fusion	Undetermined	NA	NA	COVID-NEG-043
CVN-43	Aptima MultiTest	STM	Independent Vali	July	New York	NEGATIVE	Panther Fusion	Undetermined	NA	NA	COVID-NEG-044
CVN-44	Aptima MultiTest	STM	Independent Vali	July	New York	NEGATIVE	Panther Fusion	Undetermined	NA	NA	COVID-NEG-045
CVN-45	Aptima MultiTest	STM	Independent Vali	July	New York	NEGATIVE	Panther Fusion	Undetermined	NA	NA	COVID-NEG-046
CVN-46	Aptima MultiTest	STM	Independent Vali	July	New York	NEGATIVE	Panther Fusion	Undetermined	NA	NA	COVID-NEG-047
CVN-47	Aptima MultiTest	STM	Independent Vali	July	New York	NEGATIVE	Panther Fusion	Undetermined	NA	NA	COVID-NEG-048
CVN-48	Aptima MultiTest	STM	Independent Vali	July	New York	NEGATIVE	Panther Fusion	Undetermined	NA	NA	COVID-NEG-049
CVN-49	Aptima MultiTest	STM	Independent Vali	July	New York	NEGATIVE	Panther Fusion	Undetermined	NA	NA	COVID-NEG-050
CVN-50	Aptima MultiTest	STM	Independent Vali	July	New York	NEGATIVE	Panther Fusion	Undetermined	NA	NA	COVID-NEG-051
CVN-51	Aptima MultiTest	STM	Independent Vali	July	New York	NEGATIVE	Panther Fusion	Undetermined	NA	NA	COVID-NEG-052
CVN-52	Aptima MultiTest	STM	Independent Vali	July	New York	NEGATIVE	Panther Fusion	Undetermined	NA	NA	COVID-NEG-053
CVN-53	Aptima MultiTest	STM	Independent Vali	July	New York	NEGATIVE	Panther Fusion	Undetermined	NA	NA	COVID-NEG-054
CVN-54	Aptima MultiTest	STM	Independent Vali	July	New York	NEGATIVE	Panther Fusion	Undetermined	NA	NA	COVID-NEG-055
CVN-55	Aptima MultiTest	STM	Independent Vali	July	New York	NEGATIVE	Panther Fusion	Undetermined	NA	NA	COVID-NEG-056
CVN-56	Aptima MultiTest	STM	Independent Vali	July	New York	NEGATIVE	Panther Fusion	Undetermined	NA	NA	COVID-NEG-057
CVN-57	Aptima MultiTest	STM	Independent Vali	July	New York	NEGATIVE	Panther Fusion	Undetermined	NA	NA	COVID-NEG-058
CVN-58	Aptima MultiTest	STM	Independent Vali	July	New York	NEGATIVE	Panther Fusion	Undetermined	NA	NA	COVID-NEG-059
CVN-59	Aptima MultiTest	STM	Independent Vali	July	New York	NEGATIVE	Panther Fusion	Undetermined	NA	NA	COVID-NEG-060
CVN-60	Aptima MultiTest	STM	Independent Vali	July	New York	NEGATIVE	Panther Fusion	Undetermined	NA	NA	COVID-NEG-061
CVP-31	Aptima MultiTest	STM	Independent Vali	July	New York	POSITIVE	Panther Fusion	Undetermined	NA	NA	COVID-POS-037
CVP-32	Aptima MultiTest	STM	Independent Vali	July	New York	POSITIVE	Panther Fusion	18.761	NA	NA	COVID-POS-038
CVP-33	Aptima MultiTest	STM	Independent Vali	July	New York	POSITIVE	Panther Fusion	29.045	NA	NA	COVID-POS-039
CVP-34	Aptima MultiTest	STM	Independent Vali	July	New York	POSITIVE	Panther Fusion	23.459	NA	NA	COVID-POS-040
CVP-35	Aptima MultiTest	STM	Independent Vali	July	New York	POSITIVE	Panther Fusion	27.451	NA	NA	COVID-POS-041
CVP-36	Aptima MultiTest	STM	Independent Vali	July	New York	POSITIVE	Panther Fusion	30.180	NA	NA	COVID-POS-042
CVP-37	Aptima MultiTest	STM	Independent Vali	July	New York	POSITIVE	Panther Fusion	24.750	NA	NA	COVID-POS-043

CVP-38	Aptima MultiTest	STM	Independent Vali	July	New York	POSITIVE	Panther Fusion	27.416	NA	NA	COVID-POS-044
CVP-39	Aptima MultiTest	STM	Independent Vali	July	New York	POSITIVE	Panther Fusion	Undetermined	NA	NA	COVID-POS-045
CVP-40	Aptima MultiTest	STM	Independent Vali	July	New York	POSITIVE	Panther Fusion	28.000	NA	NA	COVID-POS-046
CVP-41	Aptima MultiTest	STM	Independent Vali	July	New York	POSITIVE	Panther Fusion	Undetermined	NA	NA	COVID-POS-047
CVP-42	Aptima MultiTest	STM	Independent Vali	July	New York	POSITIVE	Panther Fusion	17.190	NA	NA	COVID-POS-048
CVP-43	Aptima MultiTest	STM	Independent Vali	July	New York	POSITIVE	Panther Fusion	15.798	NA	NA	COVID-POS-049
CVP-44	Aptima MultiTest	STM	Independent Vali	July	New York	POSITIVE	Panther Fusion	Undetermined	NA	NA	COVID-POS-050
CVP-45	Aptima MultiTest	STM	Independent Vali	July	New York	POSITIVE	Panther Fusion	Undetermined	NA	NA	COVID-POS-051
CVP-46	Aptima MultiTest	STM	Independent Vali	July	New York	POSITIVE	Panther Fusion	Undetermined	NA	NA	COVID-POS-052
CVP-47	Aptima MultiTest	STM	Independent Vali	July	New York	POSITIVE	Panther Fusion	Undetermined	NA	NA	COVID-POS-053
CVP-48	Aptima MultiTest	STM	Independent Vali	July	New York	POSITIVE	Panther Fusion	29.809	NA	NA	COVID-POS-054
CVP-49	Aptima MultiTest	STM	Independent Vali	July	New York	POSITIVE	Panther Fusion	22.164	NA	NA	COVID-POS-055
CVP-50	Aptima MultiTest	STM	Independent Vali	July	New York	POSITIVE	Panther Fusion	26.916	NA	NA	COVID-POS-056
CVP-51	Aptima MultiTest	STM	Independent Vali	July	New York	POSITIVE	Panther Fusion	29.363	NA	NA	COVID-POS-057
CVP-52	Aptima MultiTest	STM	Independent Vali	July	New York	POSITIVE	Panther Fusion	28.931	NA	NA	COVID-POS-058
CVP-53	Aptima MultiTest	STM	Independent Vali	July	New York	POSITIVE	Panther Fusion	35.437	NA	NA	COVID-POS-059
CVP-54	Aptima MultiTest	STM	Independent Vali	July	New York	POSITIVE	Panther Fusion	Undetermined	NA	NA	COVID-POS-060
CVP-55	Aptima MultiTest	STM	Independent Vali	July	New York	POSITIVE	Panther Fusion	25.584	NA	NA	COVID-POS-061
CVP-56	Aptima MultiTest	STM	Independent Vali	July	New York	POSITIVE	Panther Fusion	Undetermined	NA	NA	COVID-POS-062
CVP-57	Aptima MultiTest	STM	Independent Vali	July	New York	POSITIVE	Panther Fusion	Undetermined	NA	NA	COVID-POS-063
CVP-58	Aptima MultiTest	STM	Independent Vali	July	New York	POSITIVE	Panther Fusion	22.904	NA	NA	COVID-POS-064
CVP-59	Aptima MultiTest	STM	Independent Vali	July	New York	POSITIVE	Panther Fusion	22.326	NA	NA	COVID-POS-065
CVP-60	Aptima MultiTest	STM	Independent Vali	July	New York	POSITIVE	Panther Fusion	27.392	NA	NA	COVID-POS-066
CVP-61	NA	NA	Geographic Valid	NA	Tennessee	POSITIVE	Roche Cobas SA	NA	19.2	19.82	COVID-Pos-067_S8_L001_R1_001
CVP-62	NA	NA	Geographic Valid	NA	Tennessee	POSITIVE	Roche Cobas SA	NA	22.14	22.78	COVID-Pos-068_S9_L001_R1_001
CVP-63	NA	NA	Geographic Valid	NA	Tennessee	POSITIVE	Roche Cobas SA	NA	31.4	33.52	COVID-Pos-069_S10_L001_R1_001
CVP-64	NA	NA	Geographic Valid	NA	Tennessee	POSITIVE	Roche Cobas SA	NA	24.51	25.36	NA
CVP-65	NA	NA	Geographic Valid	NA	Tennessee	POSITIVE	Roche Cobas SA	NA	33.43	36.7	COVID-Pos-071_S11_L001_R1_001
CVP-66	NA	NA	Geographic Valid	NA	Tennessee	POSITIVE	Roche Cobas SA	NA	20.7	21.54	COVID-Pos-072_S12_L001_R1_001
CVP-67	NA	NA	Geographic Valid	NA	Tennessee	POSITIVE	Roche Cobas SA	NA	25.32	26.43	COVID-Pos-073_S13_L001_R1_001
CVP-68	NA	NA	Geographic Valid	NA	Tennessee	POSITIVE	Roche Cobas SA	NA	20.76	21.29	COVID-Pos-074_S14_L001_R1_001
CVP-69	NA	NA	Geographic Valid	NA	Tennessee	POSITIVE	Roche Cobas SA	NA	18.31	19.02	NA
CVP-70	NA	NA	Geographic Valid	NA	Tennessee	POSITIVE	Roche Cobas SA	NA	31.2	33.16	COVID-Pos-076_S15_L001_R1_001
CVP-71	NA	NA	Geographic Valid	NA	Tennessee	POSITIVE	Roche Cobas SA	NA	30.9	33.2	COVID-Pos-077_S16_L001_R1_001
CVP-72	NA	NA	Geographic Valid	NA	Tennessee	POSITIVE	Roche Cobas SA	NA	32.21	34.5	COVID-Pos-078_S17_L001_R1_001
CVP-73	NA	NA	Geographic Valid	NA	Tennessee	POSITIVE	Roche Cobas SA	NA	19.81	20.61	COVID-Pos-079_S18_L001_R1_001
CVP-74	NA	NA	Geographic Valid	NA	Tennessee	POSITIVE	Roche Cobas SA	NA	29.02	30.66	COVID-Pos-080_S19_L001_R1_001
CVP-75	NA	NA	Geographic Valid	NA	Tennessee	POSITIVE	Roche Cobas SA	NA	18	18.63	COVID-Pos-081_S20_L001_R1_001
CVP-76	NA	NA	Geographic Valid	NA	Tennessee	POSITIVE	Roche Cobas SA	NA	17.31	17.8	NA
CVP-77	NA	NA	Geographic Valid	NA	Tennessee	POSITIVE	Roche Cobas SA	NA	30.86	33.33	COVID-Pos-083_S21_L001_R1_001
CVP-78	NA	NA	Geographic Valid	NA	Tennessee	POSITIVE	Roche Cobas SA	NA	29.83	31.21	COVID-Pos-084_S22_L001_R1_001
CVP-79	NA	NA	Geographic Valid	NA	Tennessee	POSITIVE	Roche Cobas SA	NA	19.52	20.19	COVID-Pos-085_S23_L001_R1_001
CVP-80	NA	NA	Geographic Valid	NA	Tennessee	POSITIVE	Roche Cobas SA	NA	31.46	34.13	COVID-Pos-086_S7_L001_R1_001

Supplemental Table 2. The calculation of presence and absence of SARS-CoV-2 viral genome in clinical validation and an independent clinical validation. We devised a metric to compute the integral under a curve created by calculating the coverage at 1X depth using a sliding window scheme (with a window size of 100 and step size of 10). This metric was then log transformed. We have set a threshold of 8.6 if samples had less than 10,000 bases on target or 9.5 if samples had more than 10,000 bases on target. Metrics were calculated using the R statistical software package (v4.0.1).

Presence: if SARS-CoV-2 was detected. We devised a metric [I1] to compute the integral under a curve created by calculating the coverage at 1X depth using a sliding window scheme (with a window size of 100 and step size of 10). This metric was then log transformed. We have set a threshold of 8.6 if samples had less than 10,000 bases on target or 9.5 if samples had more than 10,000 bases on target. Metrics were calculated using the R statistical software package (v4.0.1). X1-X500: percent coverage at 1X-500X depths, used internally to inform the analysis. E1: evenness, used internally to inform the analysis. I1: integral. The following HS metrics are reported: bait territory, percent of selected bases, target territory, genome size, total reads and on target bases. Red indicates deviant results. Additionally, the number of reads mapped to the human genome will provide an internal control in each individual sample to be called valid.

Specimen_ID	TYPE_PCR	presence	clade	reads	X1	X5	X10	X100	E1	I1	validity	BAIT_TERRITO	PCT_SELECTED	TARGET_TERRITO	GENOME_SIZE	TOTAL_READS	ON_TARGET_E	HUMAN_ ON_	Biotia_ID
CVN-1	NEGATIVE	FALSE	NA	2473492	0.01513224	0.008436558	0.006026113	0	0	6.123732516	VALID	29869	9.00E-06	29870	3259356417	2467880	2995	226429263	COVID-NEG-002_S6_L001_R1_001
CVN-2	NEGATIVE	FALSE	NA	3252564	0.00167392	0	0	0	0	3.922073341	VALID	29869	0	29870	3259356417	3247611	11	298210227	COVID-NEG-003_S7_L001_R1_001
CVN-3	NEGATIVE	FALSE	NA	2288625	0.002343488	0.002343488	0.002343488	0	0	4.258545578	VALID	29869	4.00E-06	29870	3259356417	2248079	1215	121345936	COVID-Neg-004_S44_L001_R1_001
CVN-4	NEGATIVE	FALSE	NA	10801026	0.19789086	0.014663542	0.009775695	0	0	8.691078209	VALID	29869	9.00E-06	29870	3259356417	10775824	11041	371203778	COVID-NEG-005_S9_L001_R1_001
CVN-5	NEGATIVE	FALSE	NA	2061968	0.046468028	0	0	0	0	7.245669477	VALID	29869	8.00E-06	29870	3259356417	2053094	1761	129756751	COVID-NEG-006_S10_L001_R1_001
CVN-6	NEGATIVE	FALSE	NA	1064413	0.170706394	0.000435219	0	0	0	8.546850057	VALID	29869	5.00E-05	29870	3259356417	1057055	6583	70875601	COVID-NEG-007_S35_L001_R1_001
CVN-7	NEGATIVE	FALSE	NA	2357502	0.046769334	0	0	0	0	7.252132695	VALID	29869	5.00E-06	29870	3259356417	2317506	1307	236724	COVID-NEG-008_S36_L001_R1_001
CVN-8	NEGATIVE	FALSE	NA	2896238	0.040274523	0	0	0	0	7.102624052	VALID	29869	4.00E-06	29870	3259356417	2893354	1215	121955798	COVID-NEG-009_S37_L001_R1_001
CVN-9	NEGATIVE	FALSE	NA	4037293	0.020187479	0.012587881	0	0	0	6.411967533	VALID	29869	6.00E-06	29870	3259356417	4033669	2806	156046426	COVID-NEG-010_S38_L001_R1_001
CVN-10	NEGATIVE	FALSE	NA	1730315	0	0	0	0	0	#NAME?	VALID	29869	0	29870	3259356417	1727355	0	143663646	COVID-Neg-011_S45_L001_R1_001
CVN-11	NEGATIVE	FALSE	NA	1452222	0.019015735	0.004954804	0.004954804	0	0	6.352171755	VALID	29869	2.00E-05	29870	3259356417	1449645	3491	106590485	COVID-NEG-012_S40_L001_R1_001
CVN-12	NEGATIVE	FALSE	NA	1814277	0.410277871	0.010411784	0.004218279	0	0	9.423333753	VALID	29869	7.80E-05	29870	3259356417	1811892	17435	142326243	COVID-NEG-013_S41_L001_R1_001
CVN-13	NEGATIVE	FALSE	NA	49438	0.041345832	0	0	0	0	7.128876585	VALID	29869	0.000283	29870	3259356417	49344	1567	3017714	COVID-NEG-014_S42_L001_R1_001
CVN-14	NEGATIVE	FALSE	NA	75993	0.062738534	0	0	0	0	7.545880799	VALID	29869	0.000296	29870	3259356417	75797	2281	4176197	COVID-NEG-015_S43_L001_R1_001
CVN-15	NEGATIVE	FALSE	NA	62897	0.056109809	0.004285236	0	0	0	7.434215617	VALID	29869	0.000327	29870	3259356417	62444	2439	4525370	COVID-NEG-016_S44_L001_R1_001
CVN-16	NEGATIVE	FALSE	NA	68069	0.031134918	0	0	0	0	6.845234922	VALID	29869	0.000167	29870	3259356417	67913	1119	5251566	COVID-NEG-017_S45_L001_R1_001
CVN-17	NEGATIVE	FALSE	NA	1580183	0.017308336	0.014194844	0.009775695	0	0	6.235809512	VALID	29869	2.40E-05	29870	3259356417	1577096	4326	70789243	COVID-NEG-018_S46_L001_R1_001
CVN-18	NEGATIVE	FALSE	NA	1110690	0	0	0	0	0	#NAME?	VALID	29869	0	29870	3259356417	1107581	0	96166744	COVID-NEG-019_S47_L001_R1_001
CVN-19	NEGATIVE	FALSE	NA	2915736	0.014864412	0	0	0	0	6.105874898	VALID	29869	3.00E-06	29870	3259356417	2913475	1183	203399293	COVID-Neg-020_S46_L001_R1_001
CVN-20	NEGATIVE	FALSE	NA	2887705	0	0	0	0	0	#NAME?	VALID	29869	0	29870	3259356417	2855998	0	208946591	COVID-Neg-021_S47_L001_R1_001
CVN-21	NEGATIVE	FALSE	NA	0	0	0	0	0	0	#NAME?	INVALID	29869 ?		29870	3259356417	0	0	0	COVID-NEG-022_S50_L001_R1_001
CVN-22	NEGATIVE	FALSE	NA	210499	0.028289253	0.015734851	0.002912621	0	0	6.749386963	VALID	29869	0.000185	29870	3259356417	209358	3504	13428920	COVID-NEG-023_S51_L001_R1_001
CVN-23	NEGATIVE	FALSE	NA	235757	0	0	0	0	0	#NAME?	VALID	29869	0	29870	3259356417	235196	0	17213687	COVID-NEG-024_S52_L001_R1_001
CVN-24	NEGATIVE	FALSE	NA	1098581	0.086039505	0.081921661	0.077870773	0.007063944	0.083980583	7.861711514	VALID	29869	0.000885	29870	3259356417	1090704	116783	92272166	COVID-Neg-025_S48_L001_R1_001
CVN-25	NEGATIVE	FALSE	NA	53559	0.04348845	0.020857047	0.003883495	0	0	7.179400353	VALID	29869	0.000974	29870	3259356417	52490	5627	1753667	COVID-NEG-026_S54_L001_R1_001
CVN-26	NEGATIVE	FALSE	NA	62180	0	0	0	0	0	#NAME?	VALID	29869	0	29870	3259356417	62023	0	3964318	COVID-NEG-027_S55_L001_R1_001
CVN-27	NEGATIVE	FALSE	NA	67953	0.011114831	0	0	0	0	5.815185305	VALID	29869	3.90E-05	29870	3259356417	67814	289	3865381	COVID-NEG-028_S56_L001_R1_001
CVN-28	NEGATIVE	FALSE	NA	64494	0.001908269	0	0	0	0	4.053101604	VALID	29869	1.50E-05	29870	3259356417	64331	114	3402919	COVID-NEG-029_S57_L001_R1_001
CVN-29	NEGATIVE	FALSE	NA	131360	0	0	0	0	0	#NAME?	VALID	29869	0	29870	3259356417	131215	0	9137168	COVID-NEG-030_S58_L001_R1_001
CVN-30	NEGATIVE	FALSE	NA	90980	0.006193505	0	0	0	0	5.230406161	VALID	29869	2.00E-05	29870	3259356417	86763	101	4071720	COVID-NEG-031_S59_L001_R1_001
CVP-1	POSITIVE	TRUE	20A	3716751	0.999866086	0.999832608	0.99979913	0.999129561	0.987187003	10.31196533	VALID	29869	0.207033	29870	3259356417	3713260	96984863	230534497	COVID-POS-006_S1_L001_R1_001
CVP-2	POSITIVE	TRUE	20C	5376358	1	1	1	0.999096083	0.982724574	10.31197876	VALID	29869	0.172542	29870	3259356417	5365099	110479051	348459749	COVID-POS-007_S2_L001_R1_001
CVP-3	POSITIVE	TRUE	20A	5422292	0.999866086	0.999866086	0.99979913	0.999296953	0.987230369	10.31196533	VALID	29869	0.297506	29870	3259356417	5417696	193149870	251751939	COVID-POS-008_S3_L001_R1_001
CVP-4	POSITIVE	TRUE	19A	3465259	0.889956478	0.841914965	0.774656846	0.030097087	0.652244169	10.19720534	VALID	29869	0.002402	29870	3259356417	3459617	908084	201234095	COVID-POS-009_S4_L001_R1_001
CVP-5	POSITIVE	TRUE	20A	1256389	0.999464345	0.998259123	0.996752595	0	0.879587892	10.31190491	VALID	29869	0.005932	29870	3259356417	1254963	919481	75421830	COVID-POS-010_S5_L001_R1_001
CVP-6	POSITIVE	TRUE	20C	80637571	1	1	1	0.99979913	0.977881264	10.31197876	VALID	29869	0.96137	29870	3259356417	49952761	5922268746	180963313	COVID-POS-011_S11_L001_R1_001
CVP-7	POSITIVE	FALSE	NA	730999	0.044593237	0	0	0	0	7.204487187	VALID	29869	1.60E-05	29870	3259356417	728300	1499	62478439	COVID-POS-012_S12_L001_R1_001
CVP-8	POSITIVE	TRUE	20C	778532	1	1	0.99979913	0.998359558	0.960818227	10.31197876	VALID	29869	0.16506	29870	3259356417	774436	15913656	48002284	COVID-POS-013_S13_L001_R1_001
CVP-9	POSITIVE	TRUE	20C	6660390	0.999866086	0.999866086	0.999866086	0.999129561	0.9875853	10.31196533	VALID	29869	0.434977	29870	3259356417	6655154	353142118	188374032	COVID-POS-014_S14_L001_R1_001
CVP-10	POSITIVE	TRUE	20C	5737861	0.999899565	0.999899565	0.999296953	0.99601607	0.90892935	10.31196869	VALID	29869	0.037967	29870	3259356417	5721633	24124077	368584192	COVID-POS-015_S15_L001_R1_001
CVP-11	POSITIVE	TRUE	20A	6243585	1	1	1	0.999096083	0.987007947	10.31197876	VALID	29869	0.503266	29870	3259356417	6236791	369722069	94636025	COVID-POS-016_S16_L001_R1_001
CVP-12	POSITIVE	TRUE	20C	14308108	1	1	0.999966522	0.999229997	0.990669198	10.31197876	VALID	29869	0.834353	29870	3259356417	14298158	1503464302	81052465	COVID-POS-017_S17_L001_R1_001
CVP-13	POSITIVE	TRUE	20C	158979	1	0.999732173	0.99936391	0.997723468	0.89413914	10.31197876	VALID	29869	0.574486	29870	3259356417	158739	11316086	4784065	COVID-POS-018_S18_L001_R1_001
CVP-14	POSITIVE	TRUE	NA	68212	0.456009374	0.084365584	0.012119183	0	0.213424841	9.525476299	VALID	29869	0.005026	29870	3259356417	68032	39061	4206422	COVID-POS-019_S19_L001_R1_001
CVP-15	POSITIVE	TRUE	20A	60695705	1	1	1	0.999832608	0.976592831	10.31197876	VALID	29869	0.995236	29870	3259356417	49950996	6339499218	14975922	COVID-POS-020_S20_L001_R1_001
CVP-16	POSITIVE	TRUE	19A	244330	0.99979913	0.999598259	0.999598259	0.997154335	0.908134069	10.31195862	VALID	29869	0.463824	29870	3259356417	244087	13267047	4964282	COVID-POS-021_S21_L001_R1_001
CVP-17	POSITIVE	TRUE	19A	1178790	0.998459993	0.997823904	0.994208236	0.555507198	0.764041492	10.31119295	VALID	29869	0.026665	29870	3259356417	1176340	3621916	98732729	COVID-POS-022_S22_L001_R1_001
CVP-18	POSITIVE	TRUE	20A	1239946	0.99979913	0.99979913	0.99979913	0.997991296	0.95662143	10.31195862	VALID	29869	0.188805	29870	3259356417	1219896	26468529	81436812	COVID-POS-023_S23_L001_R1_001
CVP-19	POSITIVE	TRUE	20A	24316832	1	1	0.99979913	0.999296953	0.986086429	10.31197876	VALID	29869	0.910195	29870	3259356417	24299551	2788286419	134469844	COVID-POS-024_S24_L001_R1_001
CVP-20	POSITIVE	TRUE	19A	1255877	0.88928691	0.878707734	0.836089722	0.10020087	0.658367319	10.19516207	VALID	29869	0.008731	29870	3259356417	1251199	1269979	112419865	COVID-POS-025_S25_L001_R1_001
CVP-21	POSITIVE	TRUE	20A	239196	0.999430867	0.999062605	0.997355206	0.217308336	0.750715821	10.31189819	VALID	29869	0.077526	29870	3259356417	238715	2192286	16427173	COVID-POS-026_S26_L001_R1_001
CVP-22	POSITIVE	TRUE	20A	33357673	1	1	1	1	0.980729025	10.31197876	VALID	29869	0.990733	29870	3259356417	33333057	4206709822	24886026	COVID-POS-027_S27_L001_R1_001
CVP-23	POSITIVE	TRUE	20C	63104	0.999263475	0.999229997	0.999229997	0.950987613	0.838411906	10.31185791	VALID	29869	0.816821	29870	3259356417	62936	6011953	885938	COVID-POS-028_S60_L001_R1_001
CVP-24	POSITIVE	TRUE	20C	13271148	1	1	1	0.999397389	0.989063743	10.31197876	VALID	29869	0.976305	29870	3259356417	13200740	1638993394	22155495	COVID-POS-029_S28_L001_R1_001
CVP-25	POSITIVE	TRUE	20C	1738765	1	1	0.999866086	0.999296953	0.990139604	10.31197876	VALID	29869	0.940133	29870	3259356417	1737643	213201349	5561486	COVID-POS-030_S29_L001_R1_001
CVP-26	POSITIVE	TRUE	19A	77888	0.993438232	0.93655842	0.703113492	0	0.759549715	10.30647705	VALID	29869	0.040781	29870	3259356417	77780	386475	5159470	COVID-POS-031_S30_L001_R1_001
CVP-27	POSITIVE	TRUE	19A	117370	0.987746903	0.836859725	0.534014061	0	0.685564111	10.30086271	VALID	29869	0.023599	29870	3259356417	117124	316600	6341409	COVID-POS-032_S31_L001_R1_001
CVP-28	POSITIVE	TRUE	20B	53540594	1	1	1	0.999899565	0.97145478	10.31197876	VALID	29869	0.989716	29870	3259356417	49959831	6206809044	29989106	COVID-POS-033_S32_L001_R1_001
CVP-29	POSITIVE	TRUE	19A	102921	0.652996317	0.162638098	0.05125544	0	0.326816204	9.88578693	VALID	29869	0.005657	29870	3259356417	102661	71097	6979518	COVID-POS-034_S33_L001_R1_001
CVP-30	POSITIVE	TRUE	19A	113558	0.994810847	0.93341145	0.714429193	0	0.749154026	10.30865193	VALID	29869	0.031868	29870	3259356417	113339	425426	7062458	COVID-POS-035_S34_L001_R1_001
PC-1: Positive cont	NA	TRUE	19A	503319	0.999832608	0.999832608	0.999832608	0.987278206	0.921772525	10.3118109	VALID	29869	0.254401	29870	3259356417	502730	15570678	3979669	Ctrl1-1e4_S63_L001_R1_001
PC-2: Positive cont	NA	TRUE	19A	47012721	1	1	1	1	0.981681207	10.31197876	VALID	29869	0.992847	29870	3259356417	46976306	5807449979	3285532	Ctrl2-1e6-1_S61_L001_R1_001
PC-3: Positive cont	NA	TRUE	19A	42266116	1	1	1	1	0.987988669	10.31197876	VALID	29869	0.714139	29870	3259356417	42210524	3640762499	191152657	Ctrl2-1e6-2_S62_L001_R1_001
IC-1: Negative inte	NA	FALSE	NA	352565	0.005323067	0	0	0	0	5.078954538	VALID	29869	6.00E-06	29870	3259356417	351931	154	2140243	HuRef-1_S64_L001_R1_001
IC-2: Negative inte	NA	FALSE	NA	10516002	0.016571811	0	0	0	0	6.214608098	VALID	29869	0	29870	3259356417	10501466	113	82856654	HuRef-2_S65_L001_R1_001
NTC-1: Negative te	NA	FALSE	NA	61	0	0	0	0	0	#NAME?	VALID	29869	0	29870	3259356417	20	0	544	NC-1_S66_L001_R1_001
NTC-2: Negative te	NA	FALSE	NA	1552	0.004954804	0.004954804	0.004954804	0	0	5.00726261	VALID	29869	0.035026	29870	3259356417	833	3404	20909	NC-2_S67_L001_R1_001

Specimen_ID	TYPE_PCR	presence	clade	reads	X1	X5	X10	X100	E1	I1	validity	BAIT_TERRITO	PCT_SELECTED	TARGET_TERRITO	GENOME_SIZE	TOTAL_READS	ON_TARGET_E	HUMAN_ ON_	Specimen_ID
CVN-31	NEGATIVE	FALSE	NA	1875341	0.084968196	0	0	0	0	7.849181984	VALID	29869	1.40E-05	29870	3259356417	1830300	5	11623695	CVN-032_S1_L001_R1_001
CVN-32	NEGATIVE	FALSE	NA	1296720	0.066555072	0.033712755	0.004318714	0	0	7.604934723	VALID	29869	6.40E-05	29870	3259356417	1293993	9100	8083275	CVN-033_S2_L001_R1_001
CVN-33	NEGATIVE	FALSE	NA	911752	0.00462002	0	0	0	0	4.937304021	VALID	29869	3.00E-06	29870	3259356417	536590	81	3228928	CVN-034_S3_L001_R1_001
CVN-34	NEGATIVE	FALSE	NA	1113198	0.012052226	0	0	0	0	5.896154367	VALID	29869	4.00E-06	29870	3259356417	769442	0	9434784	CVN-035_S4_L001_R1_001
CVN-35_1	NEGATIVE	FALSE	NA	1007799	0.002979578	0	0	0	0	4.498686706	VALID	29869	1.00E-06	29870	3259356417	978437	0	27663021	CVN-036-1_S31_L001_R1_001
CVN-35_2	NEGATIVE	FALSE	NA	307574	0.019015735	0	0	0	0	6.352171755	VALID	29869	1.80E-05	29870	3259356417	302883	0	6423988	CVN-036-2_S5_L001_R1_001
CVN-36	NEGATIVE	FALSE	NA	390105	0.010311349	0	0	0	0	5.740150119	VALID	29869	9.00E-06	29870	3259356417	325086	84	4773953	CVN-037_S6_L001_R1_001
CVN-37	NEGATIVE	FALSE	NA	317484	0.006461332	0	0	0	0	5.272740525	VALID	29869	5.00E-06	29870	3259356417	317040	0	2836082	CVN-038_S7_L001_R1_001
CVN-38	NEGATIVE	FALSE	NA	1263795	0.012018748	0	0	0	0	5.893372724	VALID	29869	4.00E-06	29870	3259356417	841854	0	2982735	CVN-039_S8_L001_R1_001
CVN-39	NEGATIVE	FALSE	NA	736275	0.002678273	0	0	0	0	4.392076971	VALID	29869	1.00E-06	29870	3259356417	566082	0	2058619	CVN-040_S9_L001_R1_001
CVN-40	NEGATIVE	FALSE	NA	3200532	0	0	0	0	0	#NAME?	VALID	29869	0	29870	3259356417	2918414	0	26239199	CVN-041_S10_L001_R1_001
CVN-41	NEGATIVE	FALSE	NA	694610	0	0	0	0	0	#NAME?	VALID	29869	0	29870	3259356417	626001	0	8915447	CVN-042_S11_L001_R1_001
CVN-42	NEGATIVE	FALSE	NA	624484	0	0	0	0	0	#NAME?	VALID	29869	0	29870	3259356417	578606	0	1517186	CVN-043_S12_L001_R1_001
CVN-43	NEGATIVE	FALSE	NA	324529	0	0	0	0	0	#NAME?	VALID	29869	0	29870	3259356417	266127	0	657999	CVN-044_S13_L001_R1_001
CVN-44	NEGATIVE	FALSE	NA	319133	0	0	0	0	0	#NAME?	VALID	29869	0	29870	3259356417	224207	0	766335	CVN-045_S14_L001_R1_001
CVN-45	NEGATIVE	FALSE	NA	291425	0	0	0	0	0	#NAME?	VALID	29869	0	29870	3259356417	239589	0	2071713	CVN-046_S15_L001_R1_001
CVN-46	NEGATIVE	FALSE	NA	3457456	0.070739873	0	0	0	0	7.665914353	VALID	29869	5.00E-06	29870	3259356417	3452574	223	27735865	CVN-047_S16_L001_R1_001
CVN-47	NEGATIVE	FALSE	NA	1325097	0.015165718	0	0	0	0	6.125942461	VALID	29869	5.00E-06	29870	3259356417	943011	168	4481973	CVN-048_S17_L001_R1_001
CVN-48	NEGATIVE	FALSE	NA	672352	0.002611316	0	0	0	0	4.366759163	VALID	29869	1.00E-06	29870	3259356417	662707	0	16961962	CVN-049_S18_L001_R1_001
CVN-49	NEGATIVE	FALSE	NA	1143275	0	0	0	0	0	#NAME?	VALID	29869	0	29870	3259356417	828594	0	7241255	CVN-050_S19_L001_R1_001
CVN-50	NEGATIVE	FALSE	NA	730951	0.004954804	0	0	0	0	5.00726261	VALID	29869	3.00E-06	29870	3259356417	465514	148	3566072	CVN-051_S20_L001_R1_001
CVN-51	NEGATIVE	FALSE	NA	3033444	0.006126548	0	0	0	0	5.219536489	VALID	29869	1.00E-06	29870	3259356417	3030022	67	20622124	CVN-052_S21_L001_R1_001
CVN-52	NEGATIVE	FALSE	NA	1129558	0	0	0	0	0	#NAME?	VALID	29869	0	29870	3259356417	1127982	0	9535006	CVN-053_S22_L001_R1_001
CVN-53	NEGATIVE	FALSE	NA	532508	0	0	0	0	0	#NAME?	VALID	29869	0	29870	3259356417	518792	0	7713289	CVN-054_S23_L001_R1_001
CVN-54	NEGATIVE	FALSE	NA	689141	0.004385671	0	0	0	0	4.885247659	VALID	29869	2.00E-06	29870	3259356417	551879	131	7320165	CVN-055_S24_L001_R1_001
CVN-55	NEGATIVE	FALSE	NA	1412365	0.026816204	0	0	0	0	6.695911283	VALID	29869	7.00E-06	29870	3259356417	1084285	0	34628025	CVN-056_S25_L001_R1_001
CVN-56	NEGATIVE	FALSE	NA	841108	0	0	0	0	0	#NAME?	VALID	29869	0	29870	3259356417	792793	0	17373896	CVN-057_S26_L001_R1_001
CVN-57	NEGATIVE	FALSE	NA	854878	0.011851356	0.010344828	0.009005691	0	0	5.879347249	VALID	29869	4.00E-05	29870	3259356417	853986	3829	12467062	CVN-058_S27_L001_R1_001
CVN-58	NEGATIVE	FALSE	NA	654913	0	0	0	0	0	#NAME?	VALID	29869	0	29870	3259356417	649394	0	11765876	CVN-059_S28_L001_R1_001
CVN-59	NEGATIVE	FALSE	NA	3178590	0.004887847	0	0	0	0	4.993656958	VALID	29869	0	29870	3259356417	3082478	60	14321919	CVN-060_S29_L001_R1_001
CVN-60	NEGATIVE	FALSE	NA	299431	0	0	0	0	0	#NAME?	VALID	29869	0	29870	3259356417	299140	0	2052778	CVN-061_S30_L001_R1_001
CVP-31	POSITIVE	TRUE	19A	7934992	0.99621694	0.988684299	0.969936391	0.06618681	0.761553968	10.30893957	VALID	29869	0.002673	29870	3259356417	5253950	1385048	10696251	CVP-037_S32_L001_R1_001
CVP-32	POSITIVE	TRUE	20A	58496123	1	1	1	0.999598259	0.980142391	10.31197876	VALID	29869	0.988396	29870	3259356417	49911604	6198541419	14209909	CVP-038_S33_L001_R1_001
CVP-33	POSITIVE	TRUE	20C	1049210	0.999330432	0.999330432	0.999330432	0.998727821	0.976431564	10.31187805	VALID	29869	0.369689	29870	3259356417	1010334	42403679	34281903	CVP-039_S34_L001_R1_001
CVP-34	POSITIVE	TRUE	20C	800031	1	0.999564781	0.999564781	0.999196518	0.98457825	10.31197876	VALID	29869	0.670266	29870	3259356417	779667	63493015	8702634	CVP-040_S35_L001_R1_001
CVP-35	POSITIVE	TRUE	20A	1602710	0.999397389	0.999296953	0.999296953	0.999029126	0.960778195	10.31189148	VALID	29869	0.291104	29870	3259356417	1219753	35726666	12543853	CVP-041_S36_L001_R1_001
CVP-36	POSITIVE	TRUE	20C	2252087	0.999866086	0.999866086	0.999866086	0.999196518	0.983391377	10.31196533	VALID	29869	0.445659	29870	3259356417	2067438	103173299	22084349	CVP-042_S37_L001_R1_001
CVP-37	POSITIVE	TRUE	20A	4507123	1	1	0.99979913	0.999296953	0.989499332	10.31197876	VALID	29869	0.930967	29870	3259356417	4503306	526532631	5097123	CVP-043_S38_L001_R1_001
CVP-38	POSITIVE	TRUE	20A	4097802	1	1	0.999866086	0.999296953	0.988165488	10.31197876	VALID	29869	0.813101	29870	3259356417	4095284	400413816	8293434	CVP-044_S39_L001_R1_001
CVP-39	POSITIVE	TRUE	20A	705925	0.999430867	0.999430867	0.999430867	0.94368932	0.823516427	10.31189819	VALID	29869	0.093386	29870	3259356417	691870	7082141	3813725	CVP-045_S40_L001_R1_001
CVP-40	POSITIVE	TRUE	19A	935100	1	0.999765651	0.999464345	0.998660864	0.983576299	10.31197876	VALID	29869	0.762808	29870	3259356417	902139	81213592	4071096	CVP-046_S41_L001_R1_001
CVP-41	POSITIVE	FALSE	NA	557045	0.232541011	0.194844325	0.136524941	0.001707399	0.200903917	8.855971569	VALID	29869	0.002148	29870	3259356417	514511	114949	9038227	CVP-047_S42_L001_R1_001
CVP-42	POSITIVE	TRUE	20C	141438966	1	1	0.999966522	0.99936391	0.976216167	10.31197876	VALID	29869	0.993891	29870	3259356417	49912226	6117593749	5336196	CVP-048_S43_L001_R1_001
CVP-43	POSITIVE	TRUE	20C	116407395	1	1	1	0.999464345	0.98370594	10.31197876	VALID	29869	0.961907	29870	3259356417	49173583	5965429667	12216323	CVP-049_S44_L001_R1_001

CVP-44	POSITIVE	TRUE	NA	1129068	0.517241379	0.501339136	0.473987278	0.025376632	0.441483093	9.655414618	VALID	29869	0.004793	29870	3259356417	1127536	583404	11883739	CVP-050_S45_L001_R1_001
CVP-45	POSITIVE	TRUE	NA	726011	0.942417141	0.934181453	0.928590559	0.399263475	0.692386251	10.25362897	VALID	29869	0.048377	29870	3259356417	563641	2743930	6370849	CVP-051_S46_L001_R1_001
CVP-46	POSITIVE	TRUE	20A	917724	0.999229997	0.99916304	0.99916304	0.997355206	0.94227858	10.31184783	VALID	29869	0.235628	29870	3259356417	701630	17158271	5157614	CVP-052_S47_L001_R1_001
CVP-47	POSITIVE	TRUE	19A	399041	0.97542685	0.917073987	0.814830934	0.026380984	0.678449142	10.28847544	VALID	29869	0.024215	29870	3259356417	363849	874650	2036881	CVP-053_S48_L001_R1_001
CVP-48	POSITIVE	TRUE	20C	926795	0.999866086	0.999866086	0.99979913	0.998828256	0.983058647	10.31196533	VALID	29869	0.700454	29870	3259356417	904391	72561910	5220193	CVP-054_S49_L001_R1_001
CVP-49	POSITIVE	TRUE	20C	43366619	1	1	0.999966522	0.99936391	0.983169083	10.31197876	VALID	29869	0.973733	29870	3259356417	43221935	5329788105	17291019	CVP-055_S50_L001_R1_001
CVP-50	POSITIVE	TRUE	20A	3058620	0.99979913	0.99979913	0.999598259	0.999029126	0.97845838	10.31195862	VALID	29869	0.768957	29870	3259356417	3055094	267046187	7969640	CVP-056_S51_L001_R1_001
CVP-51	POSITIVE	TRUE	20A	1057279	0.999598259	0.999564781	0.999397389	0.967994643	0.841212748	10.31193176	VALID	29869	0.084669	29870	3259356417	829267	7195136	5597056	CVP-057_S52_L001_R1_001
CVP-52	POSITIVE	TRUE	20A	787803	0.999899565	0.999899565	0.99979913	0.998727821	0.940008698	10.31196869	VALID	29869	0.207376	29870	3259356417	786327	18415321	20370353	CVP-058_S53_L001_R1_001
CVP-53	POSITIVE	TRUE	NA	1470681	0.997656512	0.995915634	0.990458654	0.435219284	0.773411044	10.31120262	VALID	29869	0.035039	29870	3259356417	870578	2898569	7170887	CVP-059_S54_L001_R1_001
CVP-54	POSITIVE	TRUE	20C	8575143	1	1	1	0.999296953	0.989947011	10.31197876	VALID	29869	0.936643	29870	3259356417	8561287	1000400795	3414156	CVP-060_S55_L001_R1_001
CVP-55	POSITIVE	TRUE	20C	4262833	1	0.999899565	0.999866086	0.999229997	0.99064237	10.31197876	VALID	29869	0.868489	29870	3259356417	4231828	441487651	7073977	CVP-061_S56_L001_R1_001
CVP-56	POSITIVE	FALSE	NA	1215073	0.293371276	0.228858386	0.133578842	0	0.2483205	9.085065862	VALID	29869	0.000654	29870	3259356417	1213440	87253	15206791	CVP-062_S57_L001_R1_001
CVP-57	POSITIVE	TRUE	NA	3340360	0.588751255	0.307700033	0.092500837	0	0.411750921	9.784908746	VALID	29869	0.000254	29870	3259356417	3337013	95419	30080017	CVP-063_S58_L001_R1_001
CVP-58	POSITIVE	TRUE	20C	1582760	0.999899565	0.999866086	0.99979913	0.999196518	0.98679912	10.31196869	VALID	29869	0.600883	29870	3259356417	1220553	83745222	4516113	CVP-064_S59_L001_R1_001
CVP-59	POSITIVE	TRUE	20C	6012828	1	0.999899565	0.999866086	0.999229997	0.989511782	10.31197876	VALID	29869	0.879426	29870	3259356417	6009518	663184278	20335132	CVP-065_S60_L001_R1_001
CVP-60	POSITIVE	TRUE	20A	1054976	0.99979913	0.99979913	0.99979913	0.999062605	0.977237661	10.31195862	VALID	29869	0.38185	29870	3259356417	898800	38402666	6181712	CVP-066_S61_L001_R1_001
PC-1: Positive cont	NA	FALSE	NA	698112	0.123602277	0.123568798	0.118379645	0.004954804	0.123568798	8.217888279	VALID	29869	0.001897	29870	3259356417	697176	141673	2650189	Ctrl1_S68_L001_R1_001
PC-2: Positive cont	NA	FALSE	NA	998649	0.012621359	0.00987613	0.008034818	0.004854369	0.00987613	5.942295523	VALID	29869	0.000375	29870	3259356417	993104	33603	9175740	Ctrl2_S69_L001_R1_001
IC-1: Negative inte	NA	FALSE	NA	698489	0.0029461	0	0	0	0	4.48738715	VALID	29869	1.00E-06	29870	3259356417	691182	0	6368559	HuRef-1_S66_L001_R1_001
IC-2: Negative inte	NA	FALSE	NA	517708	0.00378306	0	0	0	0	4.737438155	VALID	29869	2.00E-06	29870	3259356417	517243	50	5200687	HuRef-2_S67_L001_R1_001
NEC-1: Negative e›	NA	FALSE	NA	743360	0	0	0	0	0	#NAME?	VALID	29869	0	29870	3259356417	738055	0	648401	VEROE6-1_S64_L001_R1_001
NEC-2: Negative e›	NA	FALSE	NA	406201	0	0	0	0	0	#NAME?	VALID	29869	0	29870	3259356417	404166	0	253776	VEROE6-2_S65_L001_R1_001
NTC-1: Negative te	NA	FALSE	NA	1848	0	0	0	0	0	#NAME?	VALID	29869	0	29870	3259356417	669	0	2701	VTM-1_S62_L001_R1_001
NTC-2: Negative te	NA	FALSE	NA	625	0.008536994	0.006528289	0.006528289	0	0	5.551313881	VALID	29869	0.491826	29870	3259356417	210	3299	2104	VTM-2_S63_L001_R1_001

Specimen_ID	TYPE_PCR	presence	clade	reads	X1	X5	X10	X100	E1	I1	validity	BAIT_TERRITO	PCT_SELECTED	TARGET_TERRITO	GENOME_SIZE	TOTAL_READS	ON_TARGET_E	HUMAN_ ON_	Specimen_ID
CVP-61	POSITIVE	TRUE	20C	33571630	1	0.999866086	0.999866086	0.999296953	0.991338043	10.31197876	VALID	29869	0.567963	29870	3259356417	33432742	2150689165	1295204804	COVID-Pos-067_S8_L001_R1_001
CVP-62	POSITIVE	TRUE	20C	17780698	0.999866086	0.99979913	0.99979913	0.999296953	0.990146098	10.31196533	VALID	29869	0.836394	29870	3259356417	17744075	1734427096	237910904	COVID-Pos-068_S9_L001_R1_001
CVP-63	POSITIVE	TRUE	19A	1690892	0.785671242	0.654536324	0.465450285	0.02122531	0.492170836	10.07195138	VALID	29869	0.002311	29870	3259356417	1687077	418481	126079525	COVID-Pos-069_S10_L001_R1_001
CVP-65	POSITIVE	TRUE	NA	11036664	0.708838299	0.078372949	0.055641111	0	0.193438232	9.970263153	VALID	29869	5.40E-05	29870	3259356417	11000217	23215	797636227	COVID-Pos-071_S11_L001_R1_001
CVP-66	POSITIVE	TRUE	20A	10865511	0.999966522	0.999330432	0.999296953	0.994676933	0.886862501	10.3119754	VALID	29869	0.015464	29870	3259356417	10829129	16824752	821795041	COVID-Pos-072_S12_L001_R1_001
CVP-67	POSITIVE	TRUE	20A	20208866	0.999866086	0.999866086	0.999866086	0.999296953	0.987152696	10.31196533	VALID	29869	0.672406	29870	3259356417	20172611	1608000009	493782111	COVID-Pos-073_S13_L001_R1_001
CVP-68	POSITIVE	TRUE	20A	19788983	1	1	0.999899565	0.999296953	0.991349276	10.31197876	VALID	29869	0.311586	29870	3259356417	19752696	753518427	1082554910	COVID-Pos-074_S14_L001_R1_001
CVP-70	POSITIVE	TRUE	NA	9407581	0.668630733	0.430733177	0.281988617	0	0.432259792	9.91047083	VALID	29869	0.000183	29870	3259356417	9357807	166720	739974962	COVID-Pos-076_S15_L001_R1_001
CVP-71	POSITIVE	TRUE	19A	2205867	0.804854369	0.753933713	0.649548042	0.001774356	0.605155675	10.09694219	VALID	29869	0.002418	29870	3259356417	2199945	566983	159934841	COVID-Pos-077_S16_L001_R1_001
CVP-72	POSITIVE	FALSE	NA	13954504	0.137529294	0.067458989	0.028757951	0	0.081921661	8.330741907	VALID	29869	1.20E-05	29870	3259356417	13925298	20530	1102343219	COVID-Pos-078_S17_L001_R1_001
CVP-73	POSITIVE	TRUE	20C	18275102	1	1	0.999866086	0.999330432	0.989121713	10.31197876	VALID	29869	0.487527	29870	3259356417	18211692	958393797	816679479	COVID-Pos-079_S18_L001_R1_001
CVP-74	POSITIVE	TRUE	20C	11726362	0.999062605	0.998091731	0.9970539	0.281787747	0.845822313	10.31179747	VALID	29869	0.001942	29870	3259356417	11664157	2221094	904594331	COVID-Pos-080_S19_L001_R1_001
CVP-75	POSITIVE	TRUE	20C	31917334	1	0.999966522	0.999866086	0.999296953	0.990308035	10.31197876	VALID	29869	0.497093	29870	3259356417	31857965	1865250906	1247675215	COVID-Pos-081_S20_L001_R1_001
CVP-77	POSITIVE	TRUE	20C	69100764	1	0.999933043	0.999933043	0.999296953	0.989627566	10.31197876	VALID	29869	0.795328	29870	3259356417	49881888	4871795517	845963785	COVID-Pos-083_S21_L001_R1_001
CVP-78	POSITIVE	TRUE	19A	14829910	0.894476063	0.844091061	0.796786073	0.004921326	0.686884834	10.20183269	VALID	29869	0.000462	29870	3259356417	14795608	788732	1117665326	COVID-Pos-084_S22_L001_R1_001
CVP-79	POSITIVE	TRUE	20A	67594318	1	0.999866086	0.999866086	0.99936391	0.988193371	10.31197876	VALID	29869	0.919325	29870	3259356417	49860131	5515803494	369420377	COVID-Pos-085_S23_L001_R1_001
CVP-80	POSITIVE	FALSE	NA	1654901	0.070907265	0.038901908	0.018044861	0	0	7.668277862	VALID	29869	7.10E-05	29870	3259356417	1651134	12638	116644448	COVID-Pos-086_S7_L001_R1_001
IC-1: Negative inte	NA	TRUE	NA	35512912	0.352494141	0	0	0	0	9.27193897	VALID	29869	3.00E-06	29870	3259356417	35394199	7094	262159671	HuRef-1_S5_L001_R1_001
IC-2: Negative inte	NA	FALSE	NA	26535394	0.03719451	0	0	0	0	7.023066125	VALID	29869	0	29870	3259356417	26450317	58	296239480	HuRef-2_S6_L001_R1_001
PC-1: Positive cont	NA	TRUE	19A	35122364	0.996752595	0.92879143	0.73886843	0	0.743445406	10.30895977	VALID	29869	0.000124	29870	3259356417	35015304	364062	266921741	Positive-1_S26_L001_R1_001
PC-2: Positive cont	NA	TRUE	19A	31556953	1	0.988282558	0.939872782	0.001707399	0.763359616	10.31197876	VALID	29869	0.00031	29870	3259356417	31400028	621482	196638925	Positive-2_S27_L001_R1_001
NTC-1: Negative te	NA	FALSE	NA	189821	0.150050218	0	0	0	0	8.41787499	VALID	29869	0.000476	29870	3259356417	187676	0	11720	VTM-1_S1_L001_R1_001
NTC-2: Negative te	NA	FALSE	NA	15268	0.011583529	0	0	0	0	5.856489111	VALID	29869	0.000372	29870	3259356417	14492	0	4094	VTM-2_S2_L001_R1_001
NEC-1: Negative e›	NA	FALSE	NA	25396952	0.082658185	0	0	0	0	7.821618825	VALID	29869	1.00E-06	29870	3259356417	23190789	191	80498653	VeroE6-1_S3_L001_R1_001
NEC-2: Negative e›	NA	TRUE	NA	31496180	0.486173418	0	0	0	0	9.592346129	VALID	29869	7.00E-06	29870	3259356417	28498542	1764	98747661	VeroE6-2_S4_L001_R1_001

Subsampling data. We subsampled each of the four FASTQs down to a maximum of 125,000 reads totaling up to 500,000 reads for the full set of four FASTQs per sample. If a FASTQ had fewer than 125,000 reads, it was not subsampled and proceeded through the

Specimen_ID	TYPE_PCR	presence	clade	reads	X1	X5	X10	X100	E1	I1	validity	BAIT_TERRITO	PCT_SELECTED	TARGET_TERRITO	GENOME_SIZE	TOTAL_READS	HUMAN_ ON_	Specimen_ID
CVN-1	NEGATIVE	FALSE	NA	2473492	0.006026113	0	0	0	0	5.203007187	VALID	29869	9.00E-06	29870	3259356417	498924	45782452	COVID-NEG-002_S6_L001_R1_001
CVN-2	NEGATIVE	FALSE	NA	3252564	0	0	0	0	0	#NAME?	VALID	29869	0	29870	3259356417	499282	45775822	COVID-NEG-003_S7_L001_R1_001
CVN-4	NEGATIVE	FALSE	NA	10801026	0.02289923	0	0	0	0	6.538008253	VALID	29869	1.10E-05	29870	3259356417	498903	17264995	COVID-NEG-005_S9_L001_R1_001
CVN-5	NEGATIVE	FALSE	NA	2061968	0.017542685	0	0	0	0	6.27154202	VALID	29869	9.00E-06	29870	3259356417	497977	31492807	COVID-NEG-006_S10_L001_R1_001
CVN-6	NEGATIVE	FALSE	NA	1064413	0.090927352	0	0	0	0	7.916965825	VALID	29869	4.90E-05	29870	3259356417	496552	33314243	COVID-NEG-007_S35_L001_R1_001
CVN-7	NEGATIVE	FALSE	NA	2357502	0.013090057	0	0	0	0	5.978757896	VALID	29869	7.00E-06	29870	3259356417	491566	51012	COVID-NEG-008_S36_L001_R1_001
CVN-8	NEGATIVE	FALSE	NA	2896238	0.004954804	0	0	0	0	5.00726261	VALID	29869	2.00E-06	29870	3259356417	499530	21038611	COVID-NEG-009_S37_L001_R1_001
CVN-9	NEGATIVE	FALSE	NA	4037293	0.010143957	0	0	0	0	5.723783141	VALID	29869	7.00E-06	29870	3259356417	499591	19370288	COVID-NEG-010_S38_L001_R1_001
CVN-10	NEGATIVE	FALSE	NA	1814277	0.154670238	0.004218279	0	0	0	8.44820032	VALID	29869	8.50E-05	29870	3259356417	499473	39233441	COVID-NEG-013_S41_L001_R1_001
CVN-11	NEGATIVE	FALSE	NA	49438	0.041345832	0	0	0	0	7.128876585	VALID	29869	0.000283	29870	3259356417	49344	3017714	COVID-NEG-014_S42_L001_R1_001
CVN-12	NEGATIVE	FALSE	NA	75993	0.062738534	0	0	0	0	7.545880799	VALID	29869	0.000296	29870	3259356417	75797	4176197	COVID-NEG-015_S43_L001_R1_001
CVN-13	NEGATIVE	FALSE	NA	62897	0.056109809	0.004285236	0	0	0	7.434215617	VALID	29869	0.000327	29870	3259356417	62444	4525370	COVID-NEG-016_S44_L001_R1_001
CVN-14	NEGATIVE	FALSE	NA	68069	0.031134918	0	0	0	0	6.845234922	VALID	29869	0.000167	29870	3259356417	67913	5251566	COVID-NEG-017_S45_L001_R1_001
CVN-15	NEGATIVE	FALSE	NA	1580183	0.014194844	0.009809173	0.002209575	0	0	6.059783791	VALID	29869	3.90E-05	29870	3259356417	499261	22421086	COVID-NEG-018_S46_L001_R1_001
CVN-16	NEGATIVE	FALSE	NA	1110690	0	0	0	0	0	#NAME?	VALID	29869	0	29870	3259356417	499158	43314118	COVID-NEG-019_S47_L001_R1_001
CVN-17	NEGATIVE	FALSE	NA	0	0	0	0	0	0	#NAME?	INVALID	29869	?	29870	3259356417	0	0	COVID-NEG-022_S50_L001_R1_001
CVN-18	NEGATIVE	FALSE	NA	210499	0.028289253	0.015734851	0.002912621	0	0	6.749386963	VALID	29869	0.000185	29870	3259356417	209358	13428920	COVID-NEG-023_S51_L001_R1_001
CVN-19	NEGATIVE	FALSE	NA	235757	0	0	0	0	0	#NAME?	VALID	29869	0	29870	3259356417	235196	17213687	COVID-NEG-024_S52_L001_R1_001
CVN-20	NEGATIVE	FALSE	NA	53559	0.04348845	0.020857047	0.003883495	0	0	7.179400353	VALID	29869	0.000974	29870	3259356417	52490	1753667	COVID-NEG-026_S54_L001_R1_001
CVN-21	NEGATIVE	FALSE	NA	62180	0	0	0	0	0	#NAME?	VALID	29869	0	29870	3259356417	62023	3964318	COVID-NEG-027_S55_L001_R1_001
CVN-22	NEGATIVE	FALSE	NA	67953	0.011114831	0	0	0	0	5.815185305	VALID	29869	3.90E-05	29870	3259356417	67814	3865381	COVID-NEG-028_S56_L001_R1_001
CVN-23	NEGATIVE	FALSE	NA	64494	0.001908269	0	0	0	0	4.053101604	VALID	29869	1.50E-05	29870	3259356417	64331	3402919	COVID-NEG-029_S57_L001_R1_001
CVN-24	NEGATIVE	FALSE	NA	131360	0	0	0	0	0	#NAME?	VALID	29869	0	29870	3259356417	131215	9137168	COVID-NEG-030_S58_L001_R1_001
CVN-25	NEGATIVE	FALSE	NA	90980	0.006193505	0	0	0	0	5.230406161	VALID	29869	2.00E-05	29870	3259356417	86763	4071720	COVID-NEG-031_S59_L001_R1_001
CVN-26	NEGATIVE	FALSE	NA	2288625	0.002343488	0.002343488	0	0	0	4.258545578	VALID	29869	7.00E-06	29870	3259356417	492215	26581444	COVID-Neg-004_S44_L001_R1_001
CVN-27	NEGATIVE	FALSE	NA	1730315	0	0	0	0	0	#NAME?	VALID	29869	0	29870	3259356417	499665	41549069	COVID-Neg-011_S45_L001_R1_001
CVN-28	NEGATIVE	FALSE	NA	2915736	0.004954804	0	0	0	0	5.00726261	VALID	29869	2.00E-06	29870	3259356417	499707	34849949	COVID-Neg-020_S46_L001_R1_001
CVN-29	NEGATIVE	FALSE	NA	2887705	0	0	0	0	0	#NAME?	VALID	29869	0	29870	3259356417	495031	36176839	COVID-Neg-021_S47_L001_R1_001
CVN-30	NEGATIVE	FALSE	NA	1098581	0.086039505	0.076096418	0.063475059	0	0.076983596	7.861711514	VALID	29869	0.000914	29870	3259356417	496906	42048978	COVID-Neg-025_S48_L001_R1_001
CVP-1	POSITIVE	TRUE	20A	3716751	0.999866086	0.99979913	0.999598259	0.998058252	0.92913347	10.31196533	VALID	29869	0.206576	29870	3259356417	499536	31036324	COVID-POS-006_S1_L001_R1_001
CVP-2	POSITIVE	TRUE	20C	5376358	1	0.999866086	0.999598259	0.997522598	0.895646176	10.31197876	VALID	29869	0.172166	29870	3259356417	498995	32404111	COVID-POS-007_S2_L001_R1_001
CVP-3	POSITIVE	TRUE	20A	5422292	0.999866086	0.999866086	0.999732173	0.998192166	0.950629349	10.31196533	VALID	29869	0.296747	29870	3259356417	499591	23259493	COVID-POS-008_S3_L001_R1_001
CVP-4	POSITIVE	TRUE	19A	3465259	0.808302645	0.43719451	0.133645798	0	0.520968642	10.10088118	VALID	29869	0.002452	29870	3259356417	499242	29062216	COVID-POS-009_S4_L001_R1_001
CVP-5	POSITIVE	TRUE	20A	1256389	0.999464345	0.982089053	0.750954135	0	0.825291414	10.31190491	VALID	29869	0.005789	29870	3259356417	499472	29990092	COVID-POS-010_S5_L001_R1_001
CVP-6	POSITIVE	TRUE	20C	80637571	1	0.999966522	0.999899565	0.999296953	0.988745606	10.31197876	VALID	29869	0.961537	29870	3259356417	499515	1794022	COVID-POS-011_S11_L001_R1_001
CVP-7	POSITIVE	FALSE	NA	730999	0.031871443	0	0	0	0	6.868615371	VALID	29869	1.70E-05	29870	3259356417	499596	42860237	COVID-POS-012_S12_L001_R1_001
CVP-8	POSITIVE	TRUE	20C	778532	1	0.99979913	0.99979913	0.997957817	0.906247204	10.31197876	VALID	29869	0.164561	29870	3259356417	497402	30854625	COVID-POS-013_S13_L001_R1_001
CVP-9	POSITIVE	TRUE	20C	6660390	0.999866086	0.999866086	0.99979913	0.998426515	0.981573699	10.31196533	VALID	29869	0.434469	29870	3259356417	499620	14145951	COVID-POS-014_S14_L001_R1_001
CVP-10	POSITIVE	TRUE	20C	5737861	0.999899565	0.997756947	0.995882156	0.295748242	0.723617956	10.31196869	VALID	29869	0.03814	29870	3259356417	498640	32129960	COVID-POS-015_S15_L001_R1_001
CVP-11	POSITIVE	TRUE	20A	6243585	1	0.999966522	0.99979913	0.998024774	0.985203392	10.31197876	VALID	29869	0.503105	29870	3259356417	499470	7608737	COVID-POS-016_S16_L001_R1_001
CVP-12	POSITIVE	TRUE	20C	14308108	1	0.999832608	0.99979913	0.998861734	0.989964987	10.31197876	VALID	29869	0.834466	29870	3259356417	499649	2780437	COVID-POS-017_S17_L001_R1_001
CVP-13	POSITIVE	TRUE	20C	158979	1	0.999732173	0.99936391	0.997723468	0.89413914	10.31197876	VALID	29869	0.574486	29870	3259356417	158739	4784065	COVID-POS-018_S18_L001_R1_001
CVP-14	POSITIVE	TRUE	NA	68212	0.456009374	0.084365584	0.012119183	0	0.213424841	9.525476299	VALID	29869	0.005026	29870	3259356417	68032	4206422	COVID-POS-019_S19_L001_R1_001
CVP-15	POSITIVE	TRUE	20A	60695705	1	1	1	0.998694342	0.989440799	10.31197876	VALID	29869	0.995203	29870	3259356417	499554	148937	COVID-POS-020_S20_L001_R1_001
CVP-16	POSITIVE	TRUE	19A	244330	0.99979913	0.999598259	0.999598259	0.997154335	0.908134069	10.31195862	VALID	29869	0.463824	29870	3259356417	244087	4964282	COVID-POS-021_S21_L001_R1_001
CVP-17	POSITIVE	TRUE	19A	1178790	0.997823904	0.988952126	0.969132909	0.094007365	0.756357034	10.31055445	VALID	29869	0.026852	29870	3259356417	499335	41931588	COVID-POS-022_S22_L001_R1_001
CVP-18	POSITIVE	TRUE	20A	1239946	0.99979913	0.99979913	0.999397389	0.991697355	0.861088641	10.31195862	VALID	29869	0.189109	29870	3259356417	492472	32919126	COVID-POS-023_S23_L001_R1_001
CVP-19	POSITIVE	TRUE	20A	24316832	1	0.99979913	0.999598259	0.998560429	0.988619299	10.31197876	VALID	29869	0.91035	29870	3259356417	499688	2763928	COVID-POS-024_S24_L001_R1_001
CVP-20	POSITIVE	TRUE	19A	1255877	0.885369936	0.792869099	0.642684968	0.001774356	0.634051724	10.19073814	VALID	29869	0.008593	29870	3259356417	499045	44827793	COVID-POS-025_S25_L001_R1_001
CVP-21	POSITIVE	TRUE	20A	239196	0.999430867	0.999062605	0.997355206	0.217308336	0.750715821	10.31189819	VALID	29869	0.077526	29870	3259356417	238715	16427173	COVID-POS-026_S26_L001_R1_001
CVP-22	POSITIVE	TRUE	20A	33357673	1	1	1	0.999062605	0.989035194	10.31197876	VALID	29869	0.990757	29870	3259356417	499612	370017	COVID-POS-027_S27_L001_R1_001
CVP-23	POSITIVE	TRUE	20C	63104	0.999263475	0.999229997	0.999229997	0.950987613	0.838411906	10.31185791	VALID	29869	0.816821	29870	3259356417	62936	885938	COVID-POS-028_S60_L001_R1_001
CVP-24	POSITIVE	TRUE	20C	13271148	1	1	0.999866086	0.999296953	0.988408923	10.31197876	VALID	29869	0.976171	29870	3259356417	497364	834162	COVID-POS-029_S28_L001_R1_001
CVP-25	POSITIVE	TRUE	20C	1738765	1	0.999933043	0.999866086	0.999096083	0.989697648	10.31197876	VALID	29869	0.940331	29870	3259356417	499707	1598091	COVID-POS-030_S29_L001_R1_001
CVP-26	POSITIVE	TRUE	19A	77888	0.993438232	0.93655842	0.703113492	0	0.759549715	10.30647705	VALID	29869	0.040781	29870	3259356417	77780	5159470	COVID-POS-031_S30_L001_R1_001
CVP-27	POSITIVE	TRUE	19A	117370	0.987746903	0.836859725	0.534014061	0	0.685564111	10.30086271	VALID	29869	0.023599	29870	3259356417	117124	6341409	COVID-POS-032_S31_L001_R1_001
CVP-28	POSITIVE	TRUE	20B	53540594	1	1	1	0.999029126	0.989184592	10.31197876	VALID	29869	0.989792	29870	3259356417	499609	291486	COVID-POS-033_S32_L001_R1_001
CVP-29	POSITIVE	TRUE	19A	102921	0.652996317	0.162638098	0.05125544	0	0.326816204	9.88578693	VALID	29869	0.005657	29870	3259356417	102661	6979518	COVID-POS-034_S33_L001_R1_001
CVP-30	POSITIVE	TRUE	19A	113558	0.994810847	0.93341145	0.714429193	0	0.749154026	10.30865193	VALID	29869	0.031868	29870	3259356417	113339	7062458	COVID-POS-035_S34_L001_R1_001
PC-1: Positive cont	NA	TRUE	19A	503319	0.999832608	0.999832608	0.999832608	0.987244727	0.92057409	10.3118109	VALID	29869	0.254289	29870	3259356417	498737	3949388	Ctrl1-1e4_S63_L001_R1_001
PC-2: Positive cont	NA	TRUE	19A	47012721	1	1	1	1	0.986290733	10.31197876	VALID	29869	0.99299	29870	3259356417	499601	36296	Ctrl2-1e6-1_S61_L001_R1_001
PC-3: Positive cont	NA	TRUE	19A	42266116	1	1	1	0.999598259	0.981953797	10.31197876	VALID	29869	0.714897	29870	3259356417	499391	2262938	Ctrl2-1e6-2_S62_L001_R1_001
IC-1: Negative inte	NA	FALSE	NA	352565	0.005323067	0	0	0	0	5.078954538	VALID	29869	6.00E-06	29870	3259356417	351931	2140243	HuRef-1_S64_L001_R1_001
IC-2: Negative inte	NA	FALSE	NA	10516002	0	0	0	0	0	#NAME?	VALID	29869	0	29870	3259356417	499346	3896584	HuRef-2_S65_L001_R1_001
NTC-1: Negative te	NA	FALSE	NA	61	0	0	0	0	0	#NAME?	VALID	29869	0	29870	3259356417	20	544	NC-1_S66_L001_R1_001
NTC-2: Negative te	NA	FALSE	NA	1552	0.004954804	0.004954804	0.004954804	0	0	5.00726261	VALID	29869	0.035026	29870	3259356417	833	20909	NC-2_S67_L001_R1_001

Supplemental Table 3. The preliminary LoD was established by testing 10-fold dilutions of SARS-CoV-2 synthetic RNA using two different synthetic controls in duplicates. The preliminary LoD was confirmed by testing triplicates of 2-fold dilutions (2560 copies/ml, 1280 copies/ml, 640 copies/ml, 320 copies/ml, 160 copies/ml, 80 copies/ml, and 40 copies/ml) by spiking the quantified heat-inactivated SARS-CoV-2 into negative respiratory clinical matrices. The LOD was determined to be 800 copies/ml. The LOD (800 copies/ml) was replicated 30 times. 29/30 (96.67%) samples were positive.

Presence: if SARS-CoV-2 was detected. We devised a metric [I1] to compute the integral under a curve created by calculating the coverage at 1X depth using a sliding window scheme (with a window size of 100 and step size of 10). This metric was then log transformed. We have set a threshold of 8.6 if samples had less than 10,000 bases on target or 9.5 if samples had more than 10,000 bases on target. Metrics were calculated using the R statistical software package (v4.0.1). X1-X500: percent coverage at 1X-500X depths, used internally to inform the analysis. E1: evenness, used internally to inform the analysis. I1: integral. The following HS metrics are reported: bait territory, percent of selected bases, target territory, genome size, total reads and on target bases. Red indicates deviant results. Additionally, the number of reads mapped to the human genome will provide an internal control in each individual sample to be called valid.

Specimen_ID	Copy_number	presence	reads	X1	X5	X10	X100	E1	I1	validity	BAIT_TERRITO	PCT_SELECTED	TARGET_TERRI	GENOME_SIZE	TOTAL_READS	HUMAN_ ON_TARGET_BASES
Ctrl1-1_S8_L001_R1_00	1,000,000 copi	TRUE	29242555	0.999832608	0.999832608	0.999832608	0.999832608	0.985538337	10.3118109	VALID	29869	0.961489	29870	3259356417	29196007	14388940
Ctrl1-1e1-1_S24_L001_	10 copies	FALSE	10419736	0.076163375	0.01469702	0.008938735	0	0	7.739785667	VALID	29869	6.00E-06	29870	3259356417	10386287	121167542
Ctrl1-1e1-2_S31_L001_	10 copies	FALSE	5404930	0.253398058	0.114094409	0.052058922	0	0.153766321	8.941866573	VALID	29869	6.40E-05	29870	3259356417	5388306	39509304
Ctrl1-1e2-1_S23_L001_	100 copies	TRUE	12682484	0.883662538	0.716538333	0.487345162	0	0.609015735	10.18794516	VALID	29869	0.000215	29870	3259356417	12638565	137696159
Ctrl1-1e2-2_S30_L001_	100 copies	TRUE	7914059	0.628054905	0.59306997	0.532909274	0.025677938	0.492902578	9.846468767	VALID	29869	0.000613	29870	3259356417	7894599	55935164
Ctrl1-1e3-1_S22_L001_	1000 copies	TRUE	9361244	0.999029126	0.994844325	0.97268162	0.07248075	0.783089615	10.3110048	VALID	29869	0.001467	29870	3259356417	9329773	193556872
Ctrl1-1e3-2_S29_L001_	1000 copies	TRUE	8974574	0.999497824	0.999296953	0.997288249	0.631235353	0.800771058	10.31147511	VALID	29869	0.003328	29870	3259356417	8953657	107550804
Ctrl1-1e4-1_S21_L001_	10,000 copies	TRUE	11424774	0.999698694	0.999698694	0.999531302	0.992835621	0.952392198	10.3116766	VALID	29869	0.016043	29870	3259356417	11334486	250479871
Ctrl1-1e4-2_S28_L001_	10,000 copies	TRUE	10661256	0.999832608	0.999832608	0.999832608	0.997957817	0.97135418	10.3118109	VALID	29869	0.026395	29870	3259356417	10629908	116229177
Ctrl1-1e5-1_S20_L001_	100,000 copies	TRUE	8260389	0.999832608	0.999832608	0.999832608	0.999263475	0.98540159	10.3118109	VALID	29869	0.126148	29870	3259356417	8233645	140795110
Ctrl1-1e5-2_S27_L001_	100,000 copies	TRUE	13357543	0.999832608	0.999832608	0.999832608	0.999665216	0.987786576	10.3118109	VALID	29869	0.262216	29870	3259356417	13320088	113717738
Ctrl1-1e6-1_S19_L001_	1,000,000 copi	TRUE	20139533	0.999832608	0.999832608	0.999832608	0.999665216	0.988589305	10.3118109	VALID	29869	0.630525	29870	3259356417	20099108	134874935
Ctrl1-1e6-2_S26_L001_	1,000,000 copi	TRUE	21207543	0.999832608	0.999832608	0.999832608	0.999665216	0.989106499	10.3118109	VALID	29869	0.513411	29870	3259356417	21161007	157192338
Ctrl1-2_S17_L001_R1_0	1,000,000 copi	TRUE	36430747	0.999832608	0.999832608	0.999832608	0.999665216	0.986268274	10.3118109	VALID	29869	0.869207	29870	3259356417	36349956	63869908
Ctrl2-1e1-1_S38_L001_	10 copies	FALSE	10061630	0.130632742	0.083495146	0.046534985	0	0.09916304	8.279294857	VALID	29869	2.40E-05	29870	3259356417	10034833	54260476
Ctrl2-1e1-2_S45_L001_	10 copies	FALSE	10320262	0.065048544	0.016839638	0	0	0	7.582038785	VALID	29869	5.00E-06	29870	3259356417	10295989	136974435
Ctrl2-1e2-1_S37_L001_	100 copies	TRUE	10734891	0.598627385	0.390927352	0.199899565	0	0.416002678	9.800402784	VALID	29869	0.000105	29870	3259356417	10704358	67888592
Ctrl2-1e2-2_S44_L001_	100 copies	TRUE	14469329	0.689019083	0.277770338	0.051456311	0	0.401606964	9.94188531	VALID	29869	4.70E-05	29870	3259356417	14434586	203914099
Ctrl2-1e3-1_S36_L001_	1000 copies	TRUE	10553630	0.985838634	0.922765316	0.757415467	0	0.715049528	10.29798401	VALID	29869	0.000415	29870	3259356417	10522237	64406976
Ctrl2-1e3-2_S43_L001_	1000 copies	TRUE	15517306	0.999096083	0.98844995	0.949079344	0.006427854	0.777965809	10.31128365	VALID	29869	0.000484	29870	3259356417	15476960	196558644
Ctrl2-1e4-1_S35_L001_	10,000 copies	TRUE	9463750	1	1	1	0.996149983	0.964455602	10.31197876	VALID	29869	0.022316	29870	3259356417	9439784	84515603
Ctrl2-1e4-2_S42_L001_	10,000 copies	TRUE	10707048	1	1	0.999866086	0.798292601	0.839881617	10.31197876	VALID	29869	0.003186	29870	3259356417	10676412	64493503
Ctrl2-1e5-1_S34_L001_	100,000 copies	TRUE	6097557	1	1	1	0.999933043	0.987300292	10.31197876	VALID	29869	0.313331	29870	3259356417	6083804	47758321
Ctrl2-1e5-2_S41_L001_	100,000 copies	TRUE	11434148	1	1	1	0.999899565	0.982747744	10.31197876	VALID	29869	0.045606	29870	3259356417	11401573	64417387
Ctrl2-1e6-1_S33_L001_	1,000,000 copi	TRUE	35898577	1	1	1	1	0.987777456	10.31197876	VALID	29869	0.768918	29870	3259356417	35829039	142123437
Ctrl2-1e6-2_S40_L001_	1,000,000 copi	TRUE	13566990	1	1	1	1	0.988473079	10.31197876	VALID	29869	0.411275	29870	3259356417	13537196	64086847
HuRef-1_S9_L001_R1_0	IC-1: Negative	FALSE	924528	0.109708738	0.049849347	0.005925678	0	0	8.097874995	VALID	29869	0.000123	29870	3259356417	921945	4243363
HuRef-2_S18_L001_R1_	IC-2: Negative	FALSE	2889969	0.119584868	0.020689655	0	0	0	8.190931278	VALID	29869	2.70E-05	29870	3259356417	2880457	18141077
NC-1_S25_L001_R1_001	NTC-1: Negativ	FALSE	10776962	0.032373619	0	0	0	0	6.884248831	VALID	29869	1.00E-06	29870	3259356417	10749411	160928072
NC-2_S32_L001_R1_001	NTC-2: Negativ	FALSE	2847967	0.004385671	0	0	0	0	4.885247659	VALID	29869	0	29870	3259356417	2839939	23969249
NC-3_S39_L001_R1_001	NTC-3: Negativ	FALSE	11229155	0.031904921	0	0	0	0	6.86966524	VALID	29869	1.00E-06	29870	3259356417	11198299	68087022
NC-4_S46_L001_R1_001	NTC-4: Negativ	FALSE	17662430	0.004318714	0	0	0	0	4.86986274	VALID	29869	0	29870	3259356417	17608283	255817281
Negative-Control-1_S7_	NTC-5: Negativ	FALSE	59151	0.151791095	0.036424506	0.033478406	0	0.041613659	8.429410167	VALID	29869	0.012513	29870	3259356417	58914	652
Negative-Control-2_S16	NTC-6: Negativ	FALSE	3722	0.014830934	0.014830934	0.014830934	0	0	6.103620106	VALID	29869	0.384921	29870	3259356417	392	10607

Specimen_ID	Copy_number	presence	reads	X1	X5	X10	X100	E1	I1	validity	BAIT_TERRITO	PCT_SELECTED	TARGET_TERRI	GENOME_SIZE	TOTAL_READS	HUMAN_ ON_TARGET_BASES
10-copies-1_S27_L001_	40_copies_per	TRUE	3635714	0.999096083	0.991965182	0.920053565	0.112688316	0.68126849	10.31182765	VALID	29869	0.003504	29870	3259356417	3530179	192878728
10-copies-2_S28_L001_	40_copies_per	TRUE	5210775	0.500903917	0.478908604	0.4579846	0.07037161	0.422484613	9.620548423	VALID	29869	0.001211	29870	3259356417	5204956	306570686
10-copies-3_S29_L001_	40_copies_per	TRUE	3228494	0.976297288	0.707532641	0.40629394	0	0.615203381	10.28872586	VALID	29869	0.000687	29870	3259356417	3226232	301819726
20-copies-1_S30_L001_	80_copies_per	TRUE	1558234	0.78339471	0.60629394	0.548878473	0.053632407	0.491863507	10.06977817	VALID	29869	0.004621	29870	3259356417	1522751	69894943
20-copies-2_S31_L001_	80_copies_per	TRUE	1697104	0.590023435	0.569199866	0.503481754	0.01238701	0.476665551	9.787067224	VALID	29869	0.002621	29870	3259356417	1694199	94566358
20-copies-3_S32_L001_	80_copies_per	TRUE	2871430	0.726247071	0.53150318	0.307499163	0	0.502025444	9.994795243	VALID	29869	0.000574	29870	3259356417	2869068	241551634
40-copies-1_S49_L001_	160_copies_pe	TRUE	1781187	0.568831604	0.517140944	0.404954804	0.006226984	0.444584868	9.748881316	VALID	29869	0.001835	29870	3259356417	1707976	80694315
40-copies-2_S50_L001_	160_copies_pe	FALSE	5101840	0.009273519	0.009273519	0.004318714	0	0	5.634067842	VALID	29869	7.00E-06	29870	3259356417	5041740	933784
40-copies-3_S51_L001_	160_copies_pe	TRUE	4084246	0.970539002	0.964847673	0.962202879	0.84137931	0.770097961	10.28380192	VALID	29869	0.016891	29870	3259356417	4079331	316512116
80-copies-1_S52_L001_	320_copies_pe	TRUE	3388994	0.745329762	0.285771677	0.06722464	0	0.41575159	10.01908689	VALID	29869	0.00023	29870	3259356417	3305524	239416545
80-copies-2_S53_L001_	320_copies_pe	FALSE	7571989	0.01975226	0.008938735	0	0	0	6.390172873	VALID	29869	3.00E-06	29870	3259356417	6925864	2255704
80-copies-3_S54_L001_	320_copies_pe	TRUE	4468662	0.993538668	0.993170405	0.985135588	0.733578842	0.774570701	10.3066229	VALID	29869	0.008243	29870	3259356417	4463267	320677316
160-copies-1_S55_L001	640_copies_pe	TRUE	2126034	0.914060931	0.907432206	0.863475059	0.097991296	0.678340896	10.22311388	VALID	29869	0.005902	29870	3259356417	2030354	80080662
160-copies-2_S56_L001	640_copies_pe	FALSE	11356658	0.032842317	0.032842317	0.032842317	0	0.032842317	6.898622795	VALID	29869	1.90E-05	29870	3259356417	11175869	1930825
160-copies-3_S57_L001	640_copies_pe	TRUE	6818268	0.999029126	0.999029126	0.998995648	0.9840308	0.855548912	10.31181422	VALID	29869	0.011152	29870	3259356417	6810495	515686369
320-copies-1_S58_L001	1280_copies_p	TRUE	1462814	0.988550385	0.98530298	0.97268162	0.268396384	0.721695295	10.30223378	VALID	29869	0.013312	29870	3259356417	1420671	77021657
320-copies-2_S59_L001	1280_copies_p	TRUE	1988161	0.999229997	0.999196518	0.999196518	0.997589555	0.920015595	10.31184783	VALID	29869	0.073709	29870	3259356417	1957593	86974382
320-copies-3_S60_L001	1280_copies_p	TRUE	3951638	0.997857382	0.997857382	0.997857382	0.884332106	0.795475555	10.31051747	VALID	29869	0.01646	29870	3259356417	3948697	242485573
640-copies-1_S61_L001	2560_copies_p	TRUE	3037201	0.959223301	0.639805825	0.222865752	0	0.639825912	10.27168115	VALID	29869	0.000502	29870	3259356417	2988926	249004313
640-copies-2_S62_L001	2560_copies_p	TRUE	9968231	0.99936391	0.99936391	0.99936391	0.998292601	0.932017419	10.31188476	VALID	29869	0.012978	29870	3259356417	9593974	496932225
640-copies-3_S63_L001	2560_copies_p	TRUE	5407242	0.999464345	0.999029126	0.996752595	0.036826247	0.830057171	10.31190491	VALID	29869	0.002273	29870	3259356417	5403938	339433386
COVID-Neg-003_S39_L0	CLINICAL_MAT	FALSE	4133163	0	0	0	0	0	#NAME?	VALID	29869	0	29870	3259356417	4050110	315197180
COVID-Neg-006_S40_L0	CLINICAL_MAT	FALSE	7347273	0.023635755	0.004017409	0	0	0	6.569665573	VALID	29869	2.00E-06	29870	3259356417	7103177	459058602
COVID-Neg-008_S41_L0	CLINICAL_MAT	FALSE	6041431	0.001774356	0.001774356	0.001774356	0	0	3.980342249	VALID	29869	2.00E-06	29870	3259356417	5920322	1564807
COVID-Neg-009_S42_L0	CLINICAL_MAT	FALSE	5030196	0	0	0	0	0	#NAME?	VALID	29869	0	29870	3259356417	5019325	292523032
COVID-Neg-014_S43_L0	CLINICAL_MAT	FALSE	1613606	0.020087044	0.01975226	0.018613994	0	0.020087044	6.406979991	VALID	29869	8.10E-05	29870	3259356417	1609830	122253956
Negative-Control-1_S25	NTC-1: Negativ	FALSE	6646	0.01134918	0.002142618	0.002142618	0.002142618	0	5.836050443	VALID	29869	0.033064	29870	3259356417	2484	51190
Negative-Control-2_S26	NTC-2: Negativ	FALSE	8108	0.014362236	0.010244392	0.010244392	0.007231336	0.010244392	6.071507255	VALID	29869	0.310643	29870	3259356417	3539	49276
VeroE6-1_S36_L001_R1	NEC-1: Negativ	FALSE	12259584	0	0	0	0	0	#NAME?	VALID	29869	0	29870	3259356417	12052879	27387434
VeroE6-2_S37_L001_R1	NEC-2: Negativ	FALSE	14591058	0	0	0	0	0	#NAME?	VALID	29869	0	29870	3259356417	14388229	24278506
VeroE6-3_S38_L001_R1	NEC-3: Negativ	FALSE	15735310	0.008938735	0	0	0	0	5.597298994	VALID	29869	0	29870	3259356417	15423512	53266016
VTM-1-Negative_S33_L(	NTC-3: Negativ	TRUE	16824	0.999765651	0.991965182	0.971108135	0	0.831348542	10.31195526	VALID	29869	0.821609	29870	3259356417	6499	45698
VTM-2-Negative_S34_L(	NTC-4: Negativ	FALSE	7856	0.003280884	0.003280884	0.003280884	0	0	4.595017815	VALID	29869	0.001854	29870	3259356417	5396	74396
VTM-3-Negative_S35_L(	NTC-5: Negativ	FALSE	38570	0	0	0	0	0	#NAME?	VALID	29869	0	29870	3259356417	29062	15525

Specimen_ID	Copy_number	presence	reads	X1	X5	X10	X100	E1	I1	validity	BAIT_TERRITO	PCT_SELECTED	TARGET_TERRI	GENOME_SIZE	TOTAL_READS	HUMAN_ ON_TARGET_BASES
200-10_S21_L001_R1_0	800_copies_pe	TRUE	19567791	0.732407097	0.55125544	0.38781386	0	0.511671409	10.00267584	VALID	29869	0.000246	29870	3259356417	11132676	122940840
200-11_S22_L001_R1_0	800_copies_pe	TRUE	1321419	0.898225644	0.816672246	0.701171744	0.001841312	0.632462337	10.20635062	VALID	29869	0.008107	29870	3259356417	776735	8384372
200-12_S23_L001_R1_0	800_copies_pe	TRUE	1179898	0.820120522	0.718848343	0.596417811	0	0.596484767	10.11401084	VALID	29869	0.005934	29870	3259356417	763835	16902585
200-13_S24_L001_R1_0	800_copies_pe	TRUE	45998887	0.565952461	0.017241379	0	0	0	9.74433414	VALID	29869	7.00E-06	29870	3259356417	37378484	881833606
200-14_S25_L001_R1_0	800_copies_pe	TRUE	10620613	0.895882156	0.440207566	0.085805156	0	0.556957929	10.20361064	VALID	29869	0.000128	29870	3259356417	10244131	98843845
200-15_S26_L001_R1_0	800_copies_pe	TRUE	32055937	0.997120857	0.996551724	0.995647807	0.737562772	0.774707368	10.30978657	VALID	29869	0.001398	29870	3259356417	30833828	118540231
200-16_S27_L001_R1_0	800_copies_pe	TRUE	1203350	0.992735186	0.97562772	0.916136592	0.009206562	0.749891688	10.30502615	VALID	29869	0.007983	29870	3259356417	1170632	4507215
200-17_S28_L001_R1_0	800_copies_pe	TRUE	12491956	0.958151992	0.629293606	0.281352528	0	0.630010044	10.27089697	VALID	29869	0.000165	29870	3259356417	11999480	107464020
200-18_S29_L001_R1_0	800_copies_pe	TRUE	11610093	0.960060261	0.599933043	0.221057918	0	0.653381319	10.27172658	VALID	29869	0.000158	29870	3259356417	11225387	96433434
200-19_S30_L001_R1_0	800_copies_pe	TRUE	15005488	0.982055574	0.851724138	0.633143622	0	0.716626594	10.29503167	VALID	29869	0.000231	29870	3259356417	14475151	171534027
200-1_S12_L001_R1_00	800_copies_pe	TRUE	17638397	0.822932708	0.662939404	0.436826247	0	0.571662389	10.11959632	VALID	29869	0.000338	29870	3259356417	9636299	67754989
200-20_S31_L001_R1_0	800_copies_pe	TRUE	15092369	0.918245732	0.630498828	0.295279545	0	0.599760071	10.22757937	VALID	29869	0.000143	29870	3259356417	14573562	157934041
200-21_S32_L001_R1_0	800_copies_pe	TRUE	15481079	0.871543355	0.727017074	0.512052226	0	0.618968865	10.17622902	VALID	29869	0.000287	29870	3259356417	10663574	112019872
200-22_S33_L001_R1_0	800_copies_pe	FALSE	8328941	0.099531302	0.024338801	0.000870439	0	0	7.996611816	VALID	29869	1.10E-05	29870	3259356417	7920723	580114419
200-23_S34_L001_R1_0	800_copies_pe	TRUE	28285717	0.18928691	0	0	0	0	8.64621916	VALID	29869	3.00E-06	29870	3259356417	20517187	543105407
200-24_S35_L001_R1_0	800_copies_pe	TRUE	1390054	0.992902578	0.969601607	0.891998661	0.000502176	0.761192947	10.3061275	VALID	29869	0.007254	29870	3259356417	992758	8708540
200-25_S36_L001_R1_0	800_copies_pe	TRUE	11118947	0.994442585	0.972380315	0.933980583	0.00063609	0.771242049	10.30715344	VALID	29869	0.00104	29870	3259356417	8499394	54170658
200-26_S37_L001_R1_0	800_copies_pe	TRUE	1161004	0.958151992	0.90756612	0.80860395	0.001272179	0.721742551	10.26980614	VALID	29869	0.007307	29870	3259356417	829972	8319691
200-27_S38_L001_R1_0	800_copies_pe	TRUE	17007769	0.860964178	0.289320388	0.038634081	0	0.468011383	10.16475324	VALID	29869	8.20E-05	29870	3259356417	12352019	282490493
200-28_S39_L001_R1_0	800_copies_pe	TRUE	21146650	0.259758955	0	0	0	0	8.966659075	VALID	29869	6.00E-06	29870	3259356417	15495553	445292801
200-29_S40_L001_R1_0	800_copies_pe	TRUE	10058214	0.98570472	0.98339471	0.981854704	0.926715768	0.827016962	10.29772526	VALID	29869	0.009916	29870	3259356417	9810839	259157734
200-2_S13_L001_R1_00	800_copies_pe	TRUE	12677581	0.548041513	0.396819551	0.223870104	0	0.421367593	9.711886712	VALID	29869	0.00018	29870	3259356417	8016402	281079062
200-30_S41_L001_R1_0	800_copies_pe	TRUE	14551071	0.986709073	0.969601607	0.914797456	0	0.763404515	10.29925975	VALID	29869	0.000576	29870	3259356417	14305969	790497424
200-3_S14_L001_R1_00	800_copies_pe	TRUE	28464812	0.248242384	0	0	0	0	8.92131059	VALID	29869	3.00E-06	29870	3259356417	28292444	645863434
200-4_S15_L001_R1_00	800_copies_pe	TRUE	18027697	0.821694007	0.64978239	0.412621359	0	0.567785403	10.1164547	VALID	29869	0.000133	29870	3259356417	17927625	225429392
200-5_S16_L001_R1_00	800_copies_pe	TRUE	13619534	0.58948778	0.145363241	0.01155005	0	0.311416137	9.783694646	VALID	29869	4.00E-05	29870	3259356417	13512783	268724949
200-6_S17_L001_R1_00	800_copies_pe	TRUE	18334099	0.20860395	0.000167392	0	0	0	8.747342448	VALID	29869	4.00E-06	29870	3259356417	18234520	415781789
200-7_S18_L001_R1_00	800_copies_pe	TRUE	17140943	0.656913291	0.111817877	0.0058922	0	0.284365584	9.892913859	VALID	29869	3.10E-05	29870	3259356417	17011770	248862820
200-8_S19_L001_R1_00	800_copies_pe	TRUE	15823930	0.763207231	0.336625377	0.092869099	0	0.478724473	10.04440834	VALID	29869	6.40E-05	29870	3259356417	15727502	300419232
200-9_S20_L001_R1_00	800_copies_pe	TRUE	1015377	0.652025444	0.56344158	0.460528959	0	0.505350458	9.885474396	VALID	29869	0.00591	29870	3259356417	629856	8206781
COVID-NEG-032_S7_L0(	CLINICAL_MAT	FALSE	24082540	0.004921326	0	0	0	0	5.000482923	VALID	29869	0	29870	3259356417	23172209	215139876
COVID-NEG-033_S8_L0(	CLINICAL_MAT	FALSE	18917582	0.145329762	0.095915634	0.040274523	0	0.126615333	8.385910351	VALID	29869	1.30E-05	29870	3259356417	18805063	251941381
COVID-NEG-034_S9_L0(	CLINICAL_MAT	FALSE	12325773	0	0	0	0	0	#NAME?	VALID	29869	0	29870	3259356417	6924478	67630669
COVID-NEG-035_S10_L(	CLINICAL_MAT	FALSE	14378979	0	0	0	0	0	#NAME?	VALID	29869	0	29870	3259356417	9823676	77295770
COVID-NEG-036_S11_L(	CLINICAL_MAT	FALSE	10888882	0	0	0	0	0	#NAME?	VALID	29869	0	29870	3259356417	10588610	316362634
VTM-1_S1_L001_R1_00	NTC-1: Negativ	FALSE	232	0	0	0	0	0	#NAME?	VALID	29869	0	29870	3259356417	171	4411
VTM-2_S2_L001_R1_00	NTC-2: Negativ	FALSE	1841	0	0	0	0	0	#NAME?	VALID	29869	0	29870	3259356417	1412	22687

VTM-3_S3_L001_R1_00	NTC-3: Negativ	FALSE	341	0	0	0	0	0	#NAME?	VALID	29869	0	29870	3259356417	170	4806
VeroE6-1_S4_L001_R1_	NEC-1: Negativ	FALSE	23958030	0	0	0	0	0	#NAME?	VALID	29869	0	29870	3259356417	23621777	45537443
VeroE6-2_S5_L001_R1_	NEC-2: Negativ	FALSE	10102253	0	0	0	0	0	#NAME?	VALID	29869	0	29870	3259356417	9994035	5395262
VeroE6-3_S6_L001_R1_	NEC-3: Negativ	FALSE	18004012	0	0	0	0	0	#NAME?	VALID	29869	0	29870	3259356417	17627828	36860317

Supplemental Table 4. In-silico studies inclusivity and exclusivity study included GISAID, NCBI Viral and a combined genome dataset. The total number of sequences with different percent identity is shown in the table. To account for potential cross-reactivity of the SARS-CoV-2 NGS hybridization primers, we aligned reads to 30 microbial genomes and the human genome along with the SARS-CoV-2 genome. All organisms were determined to have no cross-reactivity with the probes used, except human coronavirus HKU1 with 3 out of 994 probes with 84.6% homology and SARS-CoV-1 with 174 out of 994 probes with >80% homology. These cross-reactive probes are not expected to have significant clinical impact due to low probe coverage and/or currently low infection rates by these organisms.

DATABASE	GISAID	NCBI Virus	Combined Genome
Date	**3/29/21**	**2/16/21**	**2/16/21**
Total Sequences Reviewed	151,323	3,603,771	31
Unique Sequences Reviewed	151,323	3,603,771	31
Sequences with 100% Mean Percent Identity	50	50,200	0
Sequences with >= 80% Mean Percent Identity	151,323	65,747	2 [NC_006577.2 (human coronavirus HKU1); 84% NC_004718 (SARS-CoV-1): >80%]
Sequences with < 80% Mean Percent Identity	0	3,538,024	29
Sequences with 0% Mean Percent Identity	0	3,538,023	29
**Pathogens in Combined Genome**	**Accession**	**Above 80%**	
Adenovirus (e.g. C1 Ad. 71)	NC_001405.1	0	
Human Metapneumovirus (hMPV)	NC_039199.1	0	
Parainfluenza virus 1	JQ901971.1	0	
Parainfluenza virus 2	NC_003443.1	0	
Parainfluenza virus 3	NC_001796.2	0	
Parainfluenza virus 4	KF483663.1	0	
Influenza A	AB284320.1	0	
Influenza B	NC_002208.1	0	
Enterovirus (e.g. EV68)	AY426531.1	0	
Respiratory syncytial virus	NC_038235.1	0	
Rhinovirus	ENA|L24917|L24917.1	0	
Haemophilus influenzae	Haemophilus_influenzae_ATCC_51907	0	
Legionella pneumophila	Legionella_pneumophila_subsp_pneumophila_ATCC_33152	0	
Mycobacterium tuberculosis	NC_000962.3	0	
Streptococcus pneumoniae	Streptococcus_pneumoniae_ATCC_700669	0	
Streptococcus pyogenes	Streptococcus_pyogenes_ATCC_12344	0	
Mycoplasma pneumoniae	NZ_CP010546.1	0	
Candida albicans	28.1,CP017629.1,CP017630.1	0	
Pseudomonas aeruginosa	Pseudomonas_aeruginosa_ATCC_9027	0	
Staphylococcus epidermidis	Staphylococcus_epidermidis_ATCC_12228	0	
Staphylococcus salivarius	Streptococcus_salivarius_ATCC_9759	0	
Human coronavirus 229E	NC_002645.1	0	
Human coronavirus OC43	NC_006213.1	0	
Human coronavirus HKU1	NC_006577.2	3	
Human coronavirus NL63	DQ445911.1	0	
MERS-coronavirus	KT006149.2	0	
Chlamydia pneumonia	NC_005043.1	0	
Pneumocystis jirovecii (PCP)	GCA_001477535.1	0	
Bordetella pertussis	CP011448.1	0	
Human SARS-CoV-1	NC_004718	174	
Human Genome	GrC38	0	

Supplemental Table 5. NGS Variant Analysis and Annotation of 72 COVID Positive Samples. We detected 641 mutations at 179 different mutation sites. Out of these mutations, we identified 55 new and 124 previously reported mutations (GISAID 11-11-2020) and 38/141 new/reported mutations (GISAID 06-23-2021). The annotation provides information on the mutation name, gene location, protein name, amino acid change (NCBI) and synonymy. The frequency of genetic variants in our sample cohort was also provided. Grantham scoring system was used to designate conservative and radical mutations. In this system the score of 100 and above calls mutations radical.

Mutation Name	Sample Frequencies	Gene	Protein_Name	AA Change NCBI	Synonymy	Grantham Score	Radical >100	Reported in GISAID (11-11-2020)	Reported in GISAID 06-23-2021)
**C222T**	2	5'UTR	NA	extragenic	NA	NA	NA	Reported	Reported
**C241T**	65	5'UTR	NA	extragenic	NA	NA	NA	Reported	Reported
**C335T**	1	Orf1ab	NSP1	R24C	non-synonymous	180	radical	Reported	Reported
**C478T**	1	Orf1ab	NSP1	I71I	synonymous	NA	NA	Reported	Reported
**C601T**	1	Orf1ab	NSP1	G112G	synonymous	NA	NA	Reported	Reported
**C683T**	1	Orf1ab	NSP1	L140L	synonymous	NA	NA	Reported	Reported
**T908C**	1	Orf1ab	NSP2	L215L	synonymous	NA	NA	New	New
**C1059T**	56	Orf1ab	NSP2	T265I	non-synonymous	89	conservative	Reported	Reported
**C1170T**	1	Orf1ab	NSP2	S302F	non-synonymous	155	radical	Reported	Reported
**C1392T**	1	Orf1ab	NSP2	S376L	non-synonymous	145	radical	Reported	Reported
**G1542T**	1	Orf1ab	NSP2	R426L	non-synonymous	102	radical	New	Reported
**A1669T**	1	Orf1ab	NSP2	K468N	non-synonymous	94	conservative	New	New
**G1820A**	1	Orf1ab	NSP2	G519S	non-synonymous	56	conservative	Reported	Reported
**A1900G**	1	Orf1ab	NSP2	R545R	synonymous	NA	NA	New	New
**C1917T**	1	Orf1ab	NSP2	T551I	non-synonymous	89	conservative	Reported	Reported
**G1942T**	1	Orf1ab	NSP2	V559V	synonymous	NA	NA	Reported	Reported
**C2048T**	1	Orf1ab	NSP2	L595L	synonymous	NA	NA	Reported	Reported
**C2106T**	1	Orf1ab	NSP2	T614I	non-synonymous	89	conservative	Reported	Reported
**G2204A**	1	Orf1ab	NSP2	E647K	non-synonymous	56	conservative	New	New
**C2363T**	1	Orf1ab	NSP2	L700F	non-synonymous	22	conservative	Reported	Reported
**G2374A**	1	Orf1ab	NSP2	L703L	synonymous	NA	NA	New	New
**C2508T**	1	Orf1ab	NSP2	P748L	non-synonymous	98	conservative	Reported	Reported
**G2632T**	1	Orf1ab	NSP2	M789I	non-synonymous	10	conservative	Reported	Reported
**C3037T**	59	Orf1ab	NSP3	F924F	synonymous	NA	NA	Reported	Reported
**G3125C**	1	Orf1ab	NSP3	D954H	non-synonymous	84	conservative	New	New
**G3248A**	1	Orf1ab	NSP3	E995K	non-synonymous	56	conservative	New	New
**C3411T**	7	Orf1ab	NSP3	A1049V	non-synonymous	64	conservative	Reported	Reported
**A3427C**	1	Orf1ab	NSP3	P1054P	synonymous	NA	NA	New	Reported
**C3486T**	1	Orf1ab	NSP3	A1074V	non-synonymous	64	conservative	New	Reported
**T3713C**	1	Orf1ab	NSP3	S1150P	non-synonymous	74	conservative	New	New
**C4113T**	4	Orf1ab	NSP3	A1283V	non-synonymous	64	conservative	Reported	Reported
**A4197G**	3	Orf1ab	NSP3	E1311G	non-synonymous	98	conservative	Reported	Reported
**T4342C**	1	Orf1ab	NSP3	I1359I	synonymous	NA	NA	New	New
**A5114G**	1	Orf1ab	NSP3	T1617A	non-synonymous	58	conservative	New	New
**C5497T**	1	Orf1ab	NSP3	C1744C	synonymous	NA	NA	Reported	Reported
**G5830A**	1	Orf1ab	NSP3	K1855K	synonymous	NA	NA	Reported	Reported
**C6070T**	1	Orf1ab	NSP3	I1935I	synonymous	NA	NA	Reported	Reported
**A6295G**	2	Orf1ab	NSP3	I2010M	non-synonymous	10	conservative	Reported	Reported
**T6394C**	7	Orf1ab	NSP3	D2043D	synonymous	NA	NA	Reported	Reported
**C6706T**	1	Orf1ab	NSP3	N2147N	synonymous	NA	NA	Reported	Reported
**C6720T**	1	Orf1ab	NSP3	T2152I	non-synonymous	89	conservative	New	Reported
**G6884A**	2	Orf1ab	NSP3	G2207S	non-synonymous	56	conservative	Reported	Reported
**G6885T**	1	Orf1ab	NSP3	G2207V	non-synonymous	109	radical	New	New
**C7113T**	1	Orf1ab	NSP3	T2283I	non-synonymous	89	conservative	Reported	Reported
**C8025T**	1	Orf1ab	NSP3	A2587V	non-synonymous	64	conservative	Reported	Reported
**C8078T**	3	Orf1ab	NSP3	P2605S	non-synonymous	74	conservative	Reported	Reported
**G8179A**	4	Orf1ab	NSP3	R2638R	synonymous	NA	NA	Reported	Reported
**C8389T**	1	Orf1ab	NSP3	N2708N	synonymous	NA	NA	Reported	Reported
**A8446T**	1	Orf1ab	NSP3	K2727N	non-synonymous	94	conservative	Reported	Reported
**C8782T**	1	Orf1ab	NSP4	S2338S	synonymous	NA	NA	Reported	Reported
**C8818T**	1	Orf1ab	NSP4	C2851C	synonymous	NA	NA	Reported	Reported
**A9409T**	1	Orf1ab	NSP4	V3048V	synonymous	NA	NA	Reported	Reported
**A9409G**	1	Orf1ab	NSP4	V3048V	synonymous	NA	NA	Reported	Reported
**G9460T**	1	Orf1ab	NSP4	M3065I	non-synonymous	10	conservative	New	New
**C9969T**	1	Orf1ab	NSP4	A3235V	non-synonymous	64	conservative	Reported	Reported
**C10039T**	1	Orf1ab	NSP4	T3258T	synonymous	NA	NA	Reported	Reported
**C10319T**	7	Orf1ab	NSP5	L3352F	non-synonymous	22	conservative	Reported	Reported
**C10340T**	1	Orf1ab	NSP5	P3359S	non-synonymous	74	conservative	Reported	Reported
**C10647T**	1	Orf1ab	NSP5	T3461I	non-synonymous	89	conservative	Reported	Reported
**G10754A**	1	Orf1ab	NSP5	A3497T	non-synonymous	58	conservative	New	New
**C10851T**	2	Orf1ab	NSP5	A3529V	non-synonymous	64	conservative	Reported	Reported
**G11083T**	2	Orf1ab	NSP5	L3606F	non-synonymous	22	conservative	Reported	Reported
**C11224T**	1	Orf1ab	NSP5	V3653V	synonymous	NA	NA	Reported	Reported
**G11335T**	1	Orf1ab	NSP5	V3690V	synonymous	NA	NA	Reported	Reported
**T11459A**	1	Orf1ab	NSP5	S3732T	non-synonymous	58	conservative	New	Reported
**A11792T**	1	Orf1ab	NSP6	K3843-STOP	non-synonymous	STOP codon	radical	New	New
**C11916T**	15	Orf1ab	NSP7/Replicase	S3884L	non-synonymous	145	radical	Reported	Reported
**G12806A**	1	Orf1ab	NSP9/Replicase	V4181I	non-synonymous	29	conservative	New	New
**T12879C**	1	Orf1ab	NSP9/Replicase	I4205T	non-synonymous	89	conservative	New	New
**T12953C**	1	Orf1ab	NSP9/Replicase	F4230L	non-synonymous	22	conservative	New	New
**C13643T**	1	Orf1ab	NSP12/RNAdepRNApol	S4460F	non-synonymous	155	radical	New	Reported
**C13821T**	2	Orf1ab	NSP12/RNAdepRNApol	L4519L	synonymous	NA	NA	Reported	Reported
**T13914C**	1	Orf1ab	NSP12/RNAdepRNApol	N4550N	synonymous	NA	NA	Reported	Reported
**T14190A**	1	Orf1ab	NSP12/RNAdepRNApol	A4642A	synonymous	NA	NA	New	Reported
**C14408T**	62	Orf1ab	NSP12/RNAdepRNApol	P4715L	non-synonymous	98	conservative	Reported	Reported
**C14708T**	1	Orf1ab	NSP12/RNAdepRNApol	A4815V	non-synonymous	64	conservative	Reported	Reported
**C14805T**	2	Orf1ab	NSP12/RNAdepRNApol	Y4847Y	synonymous	NA	NA	Reported	Reported
**G15444T**	1	Orf1ab	NSP12/RNAdepRNApol	M5060I	non-synonymous	10	conservative	Reported	Reported
**T15539C**	1	Orf1ab	NSP12/RNAdepRNApol	V5092A	non-synonymous	64	conservative	New	New
**G15906T**	2	Orf1ab	NSP12/RNAdepRNApol	Q5214H	non-synonymous	24	conservative	Reported	Reported
**C15924T**	4	Orf1ab	NSP12/RNAdepRNApol	Y5220Y	synonymous	NA	NA	Reported	Reported
**C16208T**	1	Orf1ab	NSP12/RNAdepRNApol	A5315V	non-synonymous	64	conservative	New	Reported
**C16260T**	1	Orf1ab	NSP13/Helicase	C5332C	synonymous	NA	NA	Reported	Reported
**C16393T**	1	Orf1ab	NSP13/Helicase	P5377S	non-synonymous	74	conservative	Reported	Reported
**T17247C**	2	Orf1ab	NSP13/Helicase	R5661R	synonymous	NA	NA	Reported	Reported
**T17543A**	1	Orf1ab	NSP13/Helicase	M5760K	non-synonymous	95	conservative	New	New
**T17549G**	1	Orf1ab	NSP13/Helicase	L5762R	non-synonymous	102	radical	New	New
**C17550A**	1	Orf1ab	NSP13/Helicase	L5762L	synonymous	NA	NA	Reported	Reported
**C17550T**	1	Orf1ab	NSP13/Helicase	L5762L	synonymous	NA	NA	Reported	Reported
**T17556A**	1	Orf1ab	NSP13/Helicase	T5764T	synonymous	NA	NA	New	New

**C17747T**	1	Orf1ab	NSP13/Helicase	P5828L	non-synonymous	98	conservative	Reported	Reported
**G17749T**	1	Orf1ab	NSP13/Helicase	A5829S	non-synonymous	99	conservative	New	Reported
**A17858G**	1	Orf1ab	NSP13/Helicase	Y5865C	non-synonymous	194	radical	Reported	Reported
**C18060T**	1	Orf1ab	NSP14/NSP11	L5932L	synonymous	NA	NA	Reported	Reported
**G18197A**	1	Orf1ab	NSP14/NSP11	R5978K	non-synonymous	26	conservative	New	New
**G18412T**	1	Orf1ab	NSP14/NSP11	V6050F	non-synonymous	50	conservative	Reported	Reported
**G18462A**	2	Orf1ab	NSP14/NSP11	P6066P	synonymous	NA	NA	Reported	Reported
**C18486T**	1	Orf1ab	NSP14/NSP11	L6074L	synonymous	NA	NA	Reported	Reported
**C18705T**	1	Orf1ab	NSP14/NSP11	D6147D	synonymous	NA	NA	Reported	Reported
**A18774G**	2	Orf1ab	NSP14/NSP11	Q6170Q	synonymous	NA	NA	Reported	Reported
**G18782T**	1	Orf1ab	NSP14/NSP11	G6173V	non-synonymous	109	radical	Reported	Reported
**T18783del**	1	Orf1ab	NSP14/NSP11	G6173G	synonymous	NA	radical	Reported	Reported
**C18807T**	1	Orf1ab	NSP14/NSP11	N6181N	synonymous	NA	NA	Reported	Reported
**C18808T**	1	Orf1ab	NSP14/NSP11	H6182Y	non-synonymous	83	conservative	New	New
**C18877T**	3	Orf1ab	NSP14/NSP11	L6205L	synonymous	NA	NA	Reported	Reported
**C18998T**	15	Orf1ab	NSP14/NSP11	A6245V	non-synonymous	64	conservative	Reported	Reported
**A19073G**	1	Orf1ab	NSP14/NSP11	D6270G	non-synonymous	94	conservative	Reported	Reported
**T19147C**	1	Orf1ab	NSP14/NSP11	Y6295H	non-synonymous	83	conservative	New	New
**C19698T**	1	Orf1ab	NSP15/NSP11	I6478I	synonymous	NA	NA	New	Reported
**C19763T**	1	Orf1ab	NSP15/NSP11	T6500I	non-synonymous	89	conservative	Reported	Reported
**T19985C**	1	Orf1ab	NSP15/EndoRNAse	F6574S	non-synonymous	155	radical	New	New
**G20005A**	1	Orf1ab	NSP15/EndoRNAse	G6581S	non-synonymous	56	conservative	Reported	Reported
**A20755C**	2	Orf1ab	NSP16/NSP13	S6831R	non-synonymous	110	radical	Reported	Reported
**C20843T**	1	Orf1ab	NSP16/NSP13	P6860L	non-synonymous	98	conservative	Reported	Reported
**A20992T**	1	Orf1ab	NSP16/NSP13	I6910F	non-synonymous	21	conservative	New	Reported
**C21017T**	1	Orf1ab	NSP16/NSP13	T6918I	non-synonymous	89	conservative	Reported	Reported
**G21485T**	1	Orf1ab	NSP16/NSP13	S7074I	non-synonymous	142	conservative	New	Reported
**C21621T**	1	S gene	Spike	T20I	non-synonymous	89	conservative	Reported	Reported
**C21789T**	1	S gene	Spike	T76I	non-synonymous	89	conservative	Reported	Reported
**G21800A**	1	S gene	Spike	D80N	non-synonymous	23	conservative	Reported	Reported
**A21949C**	1	S gene	Spike	K129N	non-synonymous	94	conservative	New	Reported
**T22089C**	1	S gene	Spike	L176P	non-synonymous	98	conservative	New	New
**T22191C**	1	S gene	Spike	I210T	non-synonymous	89	conservative	Reported	Reported
**C22281T**	1	S gene	Spike	T240I	non-synonymous	89	conservative	Reported	Reported
**G22344C**	1	S gene	Spike	G261A	non-synonymous	60	conservative	Reported	Reported
**C22419T**	1	S gene	Spike	T286I	non-synonymous	89	conservative	New	New
**G22599A**	1	S gene	Spike/RBD	R356K	non-synonymous	26	conservative	Reported	Reported
**T22721C**	1	S gene	Spike/RBD	L387L	synonymous	NA	NA	New	New
**G22918T**	1	S gene	Spike/RBD/ACE2	L452L	synonymous	NA	NA	Reported	Reported
**T23020C**	1	S gene	Spike/RBD/ACE2	F486F	synonymous	NA	NA	New	New
**T23119C**	1	S gene	Spike/RBD	H519H	synonymous	NA	NA	New	New
**T23227C**	1	S gene	Spike/RBD	S555S	synonymous	na	NA	New	New
**A23403G**	65	S gene	Spike	D614G	non-synonymous	94	conservative	Reported	Reported
**A23723T**	1	S gene	Spike/S2	S721C	non-synonymous	112	radical	New	New
**C24270T**	1	S gene	Spike/S2/S2'	A903V	non-synonymous	64	conservative	New	New
**G24368T**	1	S gene	Spike/S2/S2'	D936Y	non-synonymous	160	radical	Reported	Reported
**T24873A**	1	S gene	Spike/S2/S2'	V1104E	non-synonymous	121	radical	New	New
**C24878A**	1	S gene	Spike/S2/S2'	Q1106K	non-synonymous	53	conservative	New	New
**G25471T**	1	Orf3a	Orf3a Protein	D27Y	non-synonymous	160	radical	Reported	Reported
**G25563T**	59	Orf3a	Orf3a Protein	Q57H	non-synonymous	24	conservative	Reported	Reported
**C25571T**	1	Orf3a	Orf3a Protein	S60F	non-synonymous	155	radical	Reported	Reported
**C25587T**	2	Orf3a	Orf3a Protein	L65L	synonymous	NA	NA	Reported	Reported
**C25613T**	1	Orf3a	Orf3a Protein	S74F	non-synonymous	155	radical	Reported	Reported
**G25690T**	1	Orf3a	Orf3a Protein	G100C	non-synonymous	159	radical	Reported	Reported
**C25693T**	1	Orf3a	Orf3a Protein	L101F	non-synonymous	22	conservative	Reported	Reported
**G25793A**	1	Orf3a	Orf3a Protein	R134H	non-synonymous	29	conservative	Reported	Reported
**A26057C**	2	Orf3a	Orf3a Protein	D222A	non-synonymous	126	radical	Reported	Reported
**G26144T**	2	Orf3a	Orf3a Protein	G251V	non-synonymous	109	radical	Reported	Reported
**G26188T**	1	Orf3a	Orf3a Protein	E266-STOP	non-synonymous	NA	radical	New	Reported
**C26425T**	1	E gene	Envelope Protein	R61C	non-synonymous	180	radical	Reported	Reported
**C26509T**	1	E/M Linking	NA	NA	NA	NA	NA	New	Reported
**G26581C**	1	M gene	Membrane/Matrix Protein	W20S	non-synonymous	177	radical	New	New
**G26992A**	1	M gene	Membrane/Matrix Protein	G157E	non-synonymous	98	conservative	New	New
**G27238T**	1	Orf6	Orf6 Protein	E13-STOP	non-synonymous	NA	radical	Reported	Reported
**G27260T**	1	Orf6	Orf6 Protein	R20M	non-synonymous	91	conservative	New	Reported
**G27506T**	1	Orf7a	Orf7a Protein	G38V	non-synonymous	109	radical	Reported	Reported
**G27915A**	1	Orf8	Orf8 Protein	G8R	non-synonymous	125	radical	Reported	Reported
**C27964T**	7	Orf8	Orf8 Protein	S24L	non-synonymous	145	radical	Reported	Reported
**G28079T**	1	Orf8	Orf8 Protein	V62V	synonymous	NA	NA	Reported	Reported
**C28093T**	1	Orf8	Orf8 Protein	S67F	non-synonymous	155	radical	Reported	Reported
**T28144C**	1	Orf8	Orf8 Protein	L84S	non-synonymous	145	radical	Reported	Reported
**C28253T**	1	Orf8	Orf8 Protein	F120F	synonymous	NA	NA	Reported	Reported
**G28326T**	1	N gene	Nucleocapsid	G18V	non-synonymous	109	radical	Reported	Reported
**C28435A**	1	N gene	Nucleocapsid	T54T	synonymous	NA	NA	Reported	Reported
**C28657T**	1	N gene	Nucleocapsid	D128D	synonymous	NA	NA	Reported	Reported
**C28821A**	1	N gene	Nucleocapsid	S183Y	non-synonymous	144	radical	Reported	Reported
**G28851T**	1	N gene	Nucleocapsid	S193I	non-synonymous	142	radical	Reported	Reported
**A28860G**	1	N gene	Nucleocapsid	N196S	non-synonymous	46	conservative	Reported	Reported
**G28881A**	2	N gene	Nucleocapsid	R203K	non-synonymous	26	conservative	Reported	Reported
**G28882A**	2	N gene	Nucleocapsid	R203R	synonymous	NA	NA	Reported	Reported
**G28883C**	2	N gene	Nucleocapsid	G204R	non-synonymous	125	radical	Reported	Reported
**C28887T**	2	N gene	Nucleocapsid	T205I	non-synonymous	89	conservative	Reported	Reported
**C29028T**	1	N gene	Nucleocapsid	A252V	non-synonymous	64	conservative	New	New
**C29280T**	1	N gene	Nucleocapsid	A336V	non-synonymous	64	conservative	New	Reported
**G29402T**	2	N gene	Nucleocapsid	D377Y	non-synonymous	160	radical	Reported	Reported
**C29445T**	1	N gene	Nucleocapsid	T391I	non-synonymous	89	conservative	Reported	Reported
**G29540A**	16	N/Orf10 Linking	NA	extragenic	NA	NA	NA	Reported	Reported
**C29686T**	1	3'UTR	NA	extragenic	NA	NA	NA	Reported	Reported
**G29779T**	1	3'UTR	NA	extragenic	NA	NA	NA	Reported	Reported

Supplemental Table 6. Validation data of SARS-CoV-2 genetic variants, *Prevotella spp.* and the primer sets. (A) To validate the variant calling of our assay and software, we selected 3 genetic regions containing mutations identified by our protocol. The genetic regions were PCR amplified using specifically designed primers followed by Sanger sequencing. The mutations for validation were selected using the following considerations: gene location, protein, synonymy, Grantham score and AA (aminoacid) replacement. Most new mutations (not yet reported by GISAID by November 11, 2020) were observed in the ORF1ab and S gene regions. One of the specific TN mutations (T6394C) was not reported by GISAID, and also included in the selection from the ORF1ab region. The primer design considered nearby mutations to the selected variants in order to maximize the validation efficiency of the sequencing reaction. The mutations that were primarily selected are highlighted in bold. (B) Metagenomic validation of *Prevotella spp.*, PCR amplification conditions and primer set.

**A. Validation of SARS-CoV-2 genetic variants and primer sets.**							
Mutations	Location	Protein	Synonymy	Grantham	AA change	Forward Primer	Reverse Primer
A6295G	ORF1ab	NSP3	non-synonymous	10	conservative	5'-ATG GTG ATG TGG TGG CTA TTG A-3'	5'-GAT CTG TGT GGC CAA CCT CT-3'
**T6394C**	ORF1ab	NSP3	synonymous	NA	NA		
**G15906T**	ORF1ab	NSP12/RNAde	non-synonymous	24	conservative	5'-GTC AAG CTG TCA CGG CCA AT-3'	5'-AAC CTG GAG CAT TGC AAA CA-3'
C15924T	ORF1ab	NSP12/RNAde pRNApol	synonymous	NA	NA		
**G21485T**	ORF1ab	NSP16/NSP13	non-synonymous	142	radical	5'-TAT CTT GGC AAA CCA CGC GA-3'	5'-CCC TGT TTT CCT TCA AGG TCC-3'
C21621T	S gene	Spike Protein	non-synonymous	89	conservative		
**B. Prevotella 16s rDNA primer set.**							
Prevotella spp.						5'-GGG ATG CGT CYG ATT AGB YWG YH-3'	5'-SCY TAG GYC GHY CCT YSC GGT-3'

Supplemental Table 7. Relevant Functional Metagenomic Profiles Obtained from HUMAnN2 Analysis. We detected a total of 434 functional pathways in our data set, of which 4 were found to be increased in CVN samples and 3 in CVP samples by LEfSE analysis. Further, we explored the functional profiles associated with the bacterial species increased in CVP and CVN samples and observed 24 pathways associated with *Actinomyces graevenitzii*, 45 with *Prevotella salivae*, 30 with *Megasphaera micronuciformis*, 28 with *Veillonella dispar*, and 11 with *Atopobium parvulum*.

Pathway ID MetaCyc	Pathway name	Taxa	COVID-Status
PWY-6168	flavin biosynthesis III (fungi)	Bacillus subtilis	CVP
PWY-6168	flavin biosynthesis III (fungi)	Dialister micraerophilus	CVP
PWY-6168	flavin biosynthesis III (fungi)	Escherichia coli	CVP
PWY-6168	flavin biosynthesis III (fungi)	Kingella denitrificans	CVP
PWY-6168	flavin biosynthesis III (fungi)	Saccharomyces cerevisiae	CVP
PWY-6168	flavin biosynthesis III (fungi)	unclassified	CVP
PWY-6936	seleno-amino acid biosynthesis	Actinomyces sp HPA0247	CVP
PWY-6936	seleno-amino acid biosynthesis	Actinomyces sp ICM39	CVP
PWY-6936	seleno-amino acid biosynthesis	Actinomyces sp ICM47	CVP
PWY-6936	seleno-amino acid biosynthesis	Bacillus subtilis	CVP
PWY-6936	seleno-amino acid biosynthesis	Campylobacter showae	CVP
PWY-6936	seleno-amino acid biosynthesis	Corynebacterium propinquum	CVP
PWY-6936	seleno-amino acid biosynthesis	Escherichia coli	CVP
PWY-6936	seleno-amino acid biosynthesis	Granulicatella adiacens	CVP
PWY-6936	seleno-amino acid biosynthesis	Haemophilus haemolyticus	CVP
PWY-6936	seleno-amino acid biosynthesis	Haemophilus parahaemolyticus	CVP
PWY-6936	seleno-amino acid biosynthesis	Haemophilus parainfluenzae	CVP
PWY-6936	seleno-amino acid biosynthesis	Haemophilus pittmaniae	CVP
PWY-6936	seleno-amino acid biosynthesis	Haemophilus sputorum	CVP
PWY-6936	seleno-amino acid biosynthesis	Kingella denitrificans	CVP
PWY-6936	seleno-amino acid biosynthesis	Lachnospiraceae bacterium ICM 7	CVP
PWY-6936	seleno-amino acid biosynthesis	Lautropia mirabilis	CVP
PWY-6936	seleno-amino acid biosynthesis	Neisseria flavescens	CVP
PWY-6936	seleno-amino acid biosynthesis	Neisseria macacae	CVP
PWY-6936	seleno-amino acid biosynthesis	Neisseria sicca	CVP
PWY-6936	seleno-amino acid biosynthesis	Neisseria sp oral taxon 14	CVP
PWY-6936	seleno-amino acid biosynthesis	Prevotella multiformis	CVP
PWY-6936	seleno-amino acid biosynthesis	Pseudomonas synxantha	CVP
PWY-6936	seleno-amino acid biosynthesis	Rothia dentocariosa	CVP
PWY-6936	seleno-amino acid biosynthesis	Rothia mucilaginosa	CVP
PWY-6936	seleno-amino acid biosynthesis	Saccharomyces cerevisiae	CVP
PWY-6936	seleno-amino acid biosynthesis	Salmonella enterica	CVP
PWY-6936	seleno-amino acid biosynthesis	Selenomonas flueggei	CVP
PWY-6936	seleno-amino acid biosynthesis	Selenomonas noxia	CVP
PWY-6936	seleno-amino acid biosynthesis	Selenomonas sputigena	CVP
PWY-6936	seleno-amino acid biosynthesis	Staphylococcus aureus	CVP
PWY-6936	seleno-amino acid biosynthesis	Staphylococcus epidermidis	CVP
PWY-6936	seleno-amino acid biosynthesis	Streptococcus infantis	CVP
PWY-6936	seleno-amino acid biosynthesis	Streptococcus mitis oralis pneumoniae	CVP
PWY-6936	seleno-amino acid biosynthesis	Streptococcus pyogenes	CVP
PWY-6936	seleno-amino acid biosynthesis	Streptococcus sanguinis	CVP
PWY-6936	seleno-amino acid biosynthesis	unclassified	CVP
PWY66-399	gluconeogenesis	unclassified	CVP
UDPNAGSYN-PWY	UDP-N-acetyl-D-glucosamine biosynthesis	Bifidobacterium dentium	CVN
UDPNAGSYN-PWY	UDP-N-acetyl-D-glucosamine biosynthesis	Bifidobacterium longum	CVN
UDPNAGSYN-PWY	UDP-N-acetyl-D-glucosamine biosynthesis	Campylobacter concisus	CVN
UDPNAGSYN-PWY	UDP-N-acetyl-D-glucosamine biosynthesis	Capnocytophaga granulosa	CVN
UDPNAGSYN-PWY	UDP-N-acetyl-D-glucosamine biosynthesis	Escherichia coli	CVN
UDPNAGSYN-PWY	UDP-N-acetyl-D-glucosamine biosynthesis	Fusobacterium nucleatum	CVN
UDPNAGSYN-PWY	UDP-N-acetyl-D-glucosamine biosynthesis	Fusobacterium periodonticum	CVN
UDPNAGSYN-PWY	UDP-N-acetyl-D-glucosamine biosynthesis	Haemophilus haemolyticus	CVN
UDPNAGSYN-PWY	UDP-N-acetyl-D-glucosamine biosynthesis	Haemophilus influenzae	CVN
UDPNAGSYN-PWY	UDP-N-acetyl-D-glucosamine biosynthesis	Lactobacillus fermentum	CVN
UDPNAGSYN-PWY	UDP-N-acetyl-D-glucosamine biosynthesis	Listeria monocytogenes	CVN
UDPNAGSYN-PWY	UDP-N-acetyl-D-glucosamine biosynthesis	Neisseria meningitidis	CVN
UDPNAGSYN-PWY	UDP-N-acetyl-D-glucosamine biosynthesis	Neisseria subflava	CVN
UDPNAGSYN-PWY	UDP-N-acetyl-D-glucosamine biosynthesis	Pseudomonas aeruginosa	CVN
UDPNAGSYN-PWY	UDP-N-acetyl-D-glucosamine biosynthesis	Pseudomonas synxantha	CVN
UDPNAGSYN-PWY	UDP-N-acetyl-D-glucosamine biosynthesis	Salmonella enterica	CVN
UDPNAGSYN-PWY	UDP-N-acetyl-D-glucosamine biosynthesis	Staphylococcus aureus	CVN
UDPNAGSYN-PWY	UDP-N-acetyl-D-glucosamine biosynthesis	Staphylococcus epidermidis	CVN
UDPNAGSYN-PWY	UDP-N-acetyl-D-glucosamine biosynthesis	Streptococcus anginosus	CVN
UDPNAGSYN-PWY	UDP-N-acetyl-D-glucosamine biosynthesis	Streptococcus constellatus	CVN
UDPNAGSYN-PWY	UDP-N-acetyl-D-glucosamine biosynthesis	Streptococcus cristatus	CVN
UDPNAGSYN-PWY	UDP-N-acetyl-D-glucosamine biosynthesis	Streptococcus gordonii	CVN
UDPNAGSYN-PWY	UDP-N-acetyl-D-glucosamine biosynthesis	Streptococcus infantis	CVN
UDPNAGSYN-PWY	UDP-N-acetyl-D-glucosamine biosynthesis	Streptococcus intermedius	CVN
UDPNAGSYN-PWY	UDP-N-acetyl-D-glucosamine biosynthesis	Streptococcus mitis oralis pneumoniae	CVN
UDPNAGSYN-PWY	UDP-N-acetyl-D-glucosamine biosynthesis	Streptococcus oligofermentans	CVN
UDPNAGSYN-PWY	UDP-N-acetyl-D-glucosamine biosynthesis	Streptococcus pseudopneumoniae	CVN
UDPNAGSYN-PWY	UDP-N-acetyl-D-glucosamine biosynthesis	Streptococcus pyogenes	CVN
UDPNAGSYN-PWY	UDP-N-acetyl-D-glucosamine biosynthesis	Streptococcus salivarius	CVN
UDPNAGSYN-PWY	UDP-N-acetyl-D-glucosamine biosynthesis	Streptococcus sanguinis	CVN
UDPNAGSYN-PWY	UDP-N-acetyl-D-glucosamine biosynthesis	Streptococcus thermophilus	CVN
UDPNAGSYN-PWY	UDP-N-acetyl-D-glucosamine biosynthesis	Streptococcus tigurinus	CVN
UDPNAGSYN-PWY	UDP-N-acetyl-D-glucosamine biosynthesis	unclassified	CVN
PWY-5030	L-histidine degradation	Fusobacterium nucleatum	CVN
PWY-5030	L-histidine degradation	Streptococcus gordonii	CVN
PWY-5030	L-histidine degradation	Streptococcus parasanguinis	CVN
PWY-5030	L-histidine degradation	Streptococcus sanguinis	CVN
PWY-5030	L-histidine degradation	unclassified	CVN
MET-SAM-PWY	superpathway of S-adenosyl-L-methionine biosynthesis	Escherichia coli	CVN
MET-SAM-PWY	superpathway of S-adenosyl-L-methionine biosynthesis	Oribacterium sinus	CVN
MET-SAM-PWY	superpathway of S-adenosyl-L-methionine biosynthesis	Salmonella enterica	CVN
MET-SAM-PWY	superpathway of S-adenosyl-L-methionine biosynthesis	Selenomonas flueggei	CVN
MET-SAM-PWY	superpathway of S-adenosyl-L-methionine biosynthesis	Streptococcus infantis	CVN
MET-SAM-PWY	superpathway of S-adenosyl-L-methionine biosynthesis	Streptococcus mitis oralis pneumoniae	CVN

MET-SAM-PWY	superpathway of S-adenosyl-L-methionine biosynthesis	Streptococcus sanguinis	CVN
MET-SAM-PWY	superpathway of S-adenosyl-L-methionine biosynthesis	unclassified	CVN
METSYN-PWY	L-homoserine and L-methionine biosynthesis	Escherichia coli	CVN
METSYN-PWY	L-homoserine and L-methionine biosynthesis	Oribacterium sinus	CVN
METSYN-PWY	L-homoserine and L-methionine biosynthesis	Salmonella enterica	CVN
METSYN-PWY	L-homoserine and L-methionine biosynthesis	Selenomonas flueggei	CVN
METSYN-PWY	L-homoserine and L-methionine biosynthesis	Streptococcus infantis	CVN
METSYN-PWY	L-homoserine and L-methionine biosynthesis	Streptococcus mitis oralis pneumoniae	CVN
METSYN-PWY	L-homoserine and L-methionine biosynthesis	Streptococcus sanguinis	CVN
METSYN-PWY	L-homoserine and L-methionine biosynthesis	unclassified	CVN
PWY-7219	adenosine ribonucleotides de novo biosynthesis	Actinomyces graevenitzii	NA
PWY-5100	pyruvate fermentation to acetate and lactate II	Actinomyces graevenitzii	NA
THRESYN-PWY	superpathway of L-threonine biosynthesis	Actinomyces graevenitzii	NA
PWY-6151	S-adenosyl-L-methionine cycle I	Actinomyces graevenitzii	NA
PWY-7221	guanosine ribonucleotides de novo biosynthesis	Actinomyces graevenitzii	NA
PWY-7111	pyruvate fermentation to isobutanol (engineered)	Actinomyces graevenitzii	NA
VALSYN-PWY	L-valine biosynthesis	Actinomyces graevenitzii	NA
PWY-6122	5-aminoimidazole ribonucleotide biosynthesis II	Actinomyces graevenitzii	NA
PWY-6277	superpathway of 5-aminoimidazole ribonucleotide biosynthesis	Actinomyces graevenitzii	NA
PWY-6121	5-aminoimidazole ribonucleotide biosynthesis I	Actinomyces graevenitzii	NA
PWY-5188	tetrapyrrole biosynthesis I (from glutamate)	Actinomyces graevenitzii	NA
PWY-7197	pyrimidine deoxyribonucleotide phosphorylation	Actinomyces graevenitzii	NA
PANTO-PWY	phosphopantothenate biosynthesis I	Actinomyces graevenitzii	NA
PWY-7208	superpathway of pyrimidine nucleobases salvage	Actinomyces graevenitzii	NA
PWY-7228	superpathway of guanosine nucleotides de novo biosynthesis I	Actinomyces graevenitzii	NA
PWY-7220	adenosine deoxyribonucleotides de novo biosynthesis II	Actinomyces graevenitzii	NA
PWY-7222	guanosine deoxyribonucleotides de novo biosynthesis II	Actinomyces graevenitzii	NA
PWY-6125	superpathway of guanosine nucleotides de novo biosynthesis II	Actinomyces graevenitzii	NA
PWY-5686	UMP biosynthesis	Actinomyces graevenitzii	NA
NONMEVIPP-PWY	methylerythritol phosphate pathway I	Actinomyces graevenitzii	NA
COA-PWY-1	coenzyme A biosynthesis II (mammalian)	Actinomyces graevenitzii	NA
PEPTIDOGLYCANSYN-PWY	peptidoglycan biosynthesis I (meso-diaminopimelate containing)	Actinomyces graevenitzii	NA
PWY-6386	UDP-N-acetylmuramoyl-pentapeptide biosynthesis II (lysine-containing)	Actinomyces graevenitzii	NA
PWY-6387	UDP-N-acetylmuramoyl-pentapeptide biosynthesis I (meso-diaminopimelate containing)	Actinomyces graevenitzii	NA
GALACTUROCAT-PWY	D-galacturonate degradation I	Prevotella salivae	NA
PWY-5989	stearate biosynthesis II (bacteria and plants)	Prevotella salivae	NA
PWY-5695	urate biosynthesis/inosine 5'-phosphate degradation	Prevotella salivae	NA
PWY-2942	L-lysine biosynthesis III	Prevotella salivae	NA
PWY-7663	gondoate biosynthesis (anaerobic)	Prevotella salivae	NA
FASYN-ELONG-PWY	fatty acid elongation -- saturated	Prevotella salivae	NA
PWY-1269	CMP-3-deoxy-D-manno-octulosonate biosynthesis I	Prevotella salivae	NA
COBALSYN-PWY	adenosylcobalamin salvage from cobinamide I	Prevotella salivae	NA
PWY-6609	adenine and adenosine salvage III	Prevotella salivae	NA
PWY-5973	cis-vaccenate biosynthesis	Prevotella salivae	NA
PWY-6282	palmitoleate biosynthesis I (from (5Z)-dodec-5-enoate)	Prevotella salivae	NA
PWY-7664	oleate biosynthesis IV (anaerobic)	Prevotella salivae	NA
PWY-6386	UDP-N-acetylmuramoyl-pentapeptide biosynthesis II (lysine-containing)	Prevotella salivae	NA
PWY-5097	L-lysine biosynthesis VI	Prevotella salivae	NA
PWY0-862	(5Z)-dodec-5-enoate biosynthesis	Prevotella salivae	NA
PEPTIDOGLYCANSYN-PWY	peptidoglycan biosynthesis I (meso-diaminopimelate containing)	Prevotella salivae	NA
ASPASN-PWY	superpathway of L-aspartate and L-asparagine biosynthesis	Prevotella salivae	NA
PWY-6387	UDP-N-acetylmuramoyl-pentapeptide biosynthesis I (meso-diaminopimelate containing)	Prevotella salivae	NA
GALACTUROCAT-PWY	D-galacturonate degradation I	Prevotella salivae	NA
PWY-7111	pyruvate fermentation to isobutanol (engineered)	Prevotella salivae	NA
VALSYN-PWY	L-valine biosynthesis	Prevotella salivae	NA
PWY-7221	guanosine ribonucleotides de novo biosynthesis	Prevotella salivae	NA
GALACT-GLUCUROCAT-PW	superpathway of hexuronide and hexuronate degradation	Prevotella salivae	NA
PWY-5686	UMP biosynthesis	Prevotella salivae	NA
PWY-6700	queuosine biosynthesis	Prevotella salivae	NA
DTDPRHAMSYN-PWY	dTDP-L-rhamnose biosynthesis I	Prevotella salivae	NA
NONMEVIPP-PWY	methylerythritol phosphate pathway I	Prevotella salivae	NA
PWY-7242	D-fructuronate degradation	Prevotella salivae	NA
PWY-6163	chorismate biosynthesis from 3-dehydroquinate	Prevotella salivae	NA
GLUCUROCAT-PWY	superpathway of β-D-glucuronide and D-glucuronate degradation	Prevotella salivae	NA
GALACT-GLUCUROCAT-PW	superpathway of hexuronide and hexuronate degradation	Prevotella salivae	NA
PWY-5667	CDP-diacylglycerol biosynthesis I	Prevotella salivae	NA
PWY0-1319	CDP-diacylglycerol biosynthesis II	Prevotella salivae	NA
COA-PWY	coenzyme A biosynthesis I	Prevotella salivae	NA
PWY-6147	6-hydroxymethyl-dihydropterin diphosphate biosynthesis I	Prevotella salivae	NA
PWY-6507	4-deoxy-L-threo-hex-4-enopyranuronate degradation	Prevotella salivae	NA
PWY-7219	adenosine ribonucleotides de novo biosynthesis	Prevotella salivae	NA
PWY-7199	pyrimidine deoxyribonucleosides salvage	Prevotella salivae	NA
PWY-6507	4-deoxy-L-threo-hex-4-enopyranuronate degradation	Prevotella salivae	NA
PWY-7242	D-fructuronate degradation	Prevotella salivae	NA
GLUCUROCAT-PWY	superpathway of β-D-glucuronide and D-glucuronate degradation	Prevotella salivae	NA
PWY-6151	S-adenosyl-L-methionine cycle I	Prevotella salivae	NA
PWY-6124	inosine-5'-phosphate biosynthesis II	Prevotella salivae	NA
PWY-6123	inosine-5'-phosphate biosynthesis I	Prevotella salivae	NA
COA-PWY-1	coenzyme A biosynthesis II (mammalian)	Prevotella salivae	NA
PWY-7111	pyruvate fermentation to isobutanol (engineered)	Megasphaera micronuciformis	NA
VALSYN-PWY	L-valine biosynthesis	Megasphaera micronuciformis	NA
PWY-6163	chorismate biosynthesis from 3-dehydroquinate	Megasphaera micronuciformis	NA
PWY-7221	guanosine ribonucleotides de novo biosynthesis	Megasphaera micronuciformis	NA
PWY-5695	urate biosynthesis/inosine 5'-phosphate degradation	Megasphaera micronuciformis	NA
COA-PWY	coenzyme A biosynthesis I	Megasphaera micronuciformis	NA
THRESYN-PWY	superpathway of L-threonine biosynthesis	Megasphaera micronuciformis	NA
PWY-7219	adenosine ribonucleotides de novo biosynthesis	Megasphaera micronuciformis	NA
PEPTIDOGLYCANSYN-PWY	peptidoglycan biosynthesis I (meso-diaminopimelate containing)	Megasphaera micronuciformis	NA
PWY-4242	pantothenate and coenzyme A biosynthesis III	Megasphaera micronuciformis	NA
PWY-6608	guanosine nucleotides degradation III	Megasphaera micronuciformis	NA
PWY-6387	UDP-N-acetylmuramoyl-pentapeptide biosynthesis I (meso-diaminopimelate containing)	Megasphaera micronuciformis	NA
COA-PWY-1	coenzyme A biosynthesis II (mammalian)	Megasphaera micronuciformis	NA

PWY-6897	thiamin salvage II	Megasphaera micronuciformis	NA
PWY-5686	UMP biosynthesis	Megasphaera micronuciformis	NA
PWY-2942	L-lysine biosynthesis III	Megasphaera micronuciformis	NA
PWY-5097	L-lysine biosynthesis VI	Megasphaera micronuciformis	NA
PWY0-1296	purine ribonucleosides degradation	Megasphaera micronuciformis	NA
PWY-7357	thiamin formation from pyrithiamine and oxythiamine (yeast)	Megasphaera micronuciformis	NA
PWY-6122	5-aminoimidazole ribonucleotide biosynthesis II	Megasphaera micronuciformis	NA
PWY-6277	superpathway of 5-aminoimidazole ribonucleotide biosynthesis	Megasphaera micronuciformis	NA
PWY-724	superpathway of L-lysine, L-threonine and L-methionine biosynthesis II	Megasphaera micronuciformis	NA
PWY-6121	5-aminoimidazole ribonucleotide biosynthesis I	Megasphaera micronuciformis	NA
PWY-5667	CDP-diacylglycerol biosynthesis I	Megasphaera micronuciformis	NA
PWY0-1319	CDP-diacylglycerol biosynthesis II	Megasphaera micronuciformis	NA
PWY-6386	UDP-N-acetylmuramoyl-pentapeptide biosynthesis II (lysine-containing)	Megasphaera micronuciformis	NA
PWY-6385	peptidoglycan biosynthesis III (mycobacteria)	Megasphaera micronuciformis	NA
PWY-6700	queuosine biosynthesis	Megasphaera micronuciformis	NA
PWY-6703	preQ0 biosynthesis	Megasphaera micronuciformis	NA
PWY-6147	6-hydroxymethyl-dihydropterin diphosphate biosynthesis I	Megasphaera micronuciformis	NA
PWY-7219	adenosine ribonucleotides de novo biosynthesis	Atopobium parvulum	NA
PWY0-1296	purine ribonucleosides degradation	Atopobium parvulum	NA
DTDPRHAMSYN-PWY	dTDP-L-rhamnose biosynthesis I	Atopobium parvulum	NA
PWY-2942	L-lysine biosynthesis III	Atopobium parvulum	NA
PWY-6609	adenine and adenosine salvage III	Atopobium parvulum	NA
PWY-5686	UMP biosynthesis	Atopobium parvulum	NA
PWY-7221	guanosine ribonucleotides de novo biosynthesis	Atopobium parvulum	NA
ASPASN-PWY	superpathway of L-aspartate and L-asparagine biosynthesis	Atopobium parvulum	NA
PWY-6386	UDP-N-acetylmuramoyl-pentapeptide biosynthesis II (lysine-containing)	Atopobium parvulum	NA
PWY0-1586	peptidoglycan maturation (meso-diaminopimelate containing)	Atopobium parvulum	NA
COA-PWY-1	coenzyme A biosynthesis II (mammalian)	Atopobium parvulum	NA
